# Modulation of SIRT3 expression through CDK4/6 enhances the anti-cancer effect of sorafenib in hepatocellular carcinoma cells

**DOI:** 10.1186/s12885-020-06822-4

**Published:** 2020-04-19

**Authors:** Hanhee Jo, Yusun Park, Taehun Kim, Jisu Kim, Jong Sook Lee, Seon Yoo Kim, Jee-in Chung, Hae yong Ko, Jae-Chul Pyun, Kyung Sik Kim, Misu Lee, Mijin Yun

**Affiliations:** 1 Division of Life Sciences, College of Life Science and Bioengineering, Incheon National University, Incheon, South Korea; 2 Department of Nuclear Medicine, Severance Hospital, Yonsei University College of Medicine, Seoul, 120-749 South Korea; 3 Department of Materials Science and Engineering, Yonsei University, Seoul, South Korea; 4 Department of Surgery, Yonsei University College of Medicine, Seoul, South Korea

**Keywords:** Hepatocellular carcinoma, SIRT3, Sorafenib, Anti-tumor effect, Drug sensitivity, CDK4/6 inhibitor

## Abstract

**Background:**

Hepatocellular carcinoma (HCC) is the leading cause of cancer-related deaths worldwide. The only drug currently approved for clinical use in the treatment of advanced HCC is sorafenib. However, many patients with HCC show reduced sensitivity to sorafenib during treatment. SIRT3, a member of the mammalian sirtuin family, is a tumor suppressor in certain tumor types. However, only few studies have investigated the effects of SIRT3 on tumor prognosis and sorafenib sensitivity in patients with HCC. Here, we aimed to investigate the correlation between SIRT3 expression and glucose metabolism and proliferation in HCC and discover effective compounds that increase endogenous SIRT3 modulation effect of sorafenib.

**Methods:**

To determine the correlation between SIRT3 and glucose related proteins, immunostaining was performed with liver cancer tissue using various antibodies. To investigate whether the expression of SIRT3 in HCC is related to the resistance to sorafenib, we treated sorafenib after the modulation of SIRT3 levels in HCC cell lines (overexpression in Huh7, knockdown in HepG2). We also employed PD0332991 to modulate the SIRT3 expression in HCC cell and conducted functional assays.

**Results:**

SIRT3 expression was downregulated in high glycolytic and proliferative HCC cells of human patients, xenograft model and HCC cell lines. Moreover, SIRT3 expression was downregulated after sorafenib treatment, resulting in reduced drug sensitivity in HCC cell lines. To enhance the anti-tumor effect of sorafenib, we employed PD0332991 (CDK4/6-Rb inhibitor) based on the correlation between SIRT3 and phosphorylated retinoblastoma protein in HCC. Notably, combined treatment with sorafenib and PD0332991 showed an enhancement of the anti-tumor effect in HCC cells.

**Conclusions:**

Our data suggest that the modulation of SIRT3 by CDK4/6 inhibition might be useful for HCC therapy together with sorafenib, which, unfortunately, has limited efficacy and whose use is often associated with drug resistance.

## Background

Hepatocellular carcinoma (HCC) is a leading cause of cancer-related deaths worldwide [1]. Patients with early-stage HCC are asymptomatic; hence, HCC is usually detected at intermediate or advanced stages, in which patients cannot receive curative treatments such as ablation, surgical resection, or liver transplantation [2]. Although surgical treatment has improved the disease outcome, the risk of recurrence remains substantial even for early HCC. In patients with advanced HCC, sorafenib (Nexavar), an orally active multikinase inhibitor, has been used as a first-line chemotherapeutic agent [3]. Despite extending the median survival by 3–5 months, the high resistance rate and serious adverse side effects have significantly limited the benefits of sorafenib therapy [4–6]. Therefore, there is an increasing need for a strategy to enhance the effects of sorafenib anti-cancer activity.

Sirtuins (SIRT1–7) have emerged as important regulators of tumorigenic processes such as proliferation, cell cycle progression, cell survival, metabolism, and angiogenesis [7–9]. SIRT3, the best characterized mitochondrial sirtuin, deacetylates and activates several enzymes involved in cellular redox balance and defense against oxidative damage [10–12]. Several reports suggest that SIRT3 has a dual role in cancer [13–15]. SIRT3 functions as an oncogene in oral cancer and melanoma by maintaining ROS levels under a certain threshold to prevent apoptosis and promote cell proliferation [16, 17]. In contrast, SIRT3 has been identified as a tumor suppressor in HCC [18, 19], breast cancer [20], ovarian cancer [21], and leukemia [22]. Further, it has been reported that SIRT3 plays a role in metabolic reprogramming (Warburg) and in triggering cell death under stress conditions [23, 24]. Indeed, high SIRT3 expression is correlated with favorable outcomes and an increase in the overall survival rate of patients with HCC [25]. In this regard, regulation of SIRT3 expression might be a novel strategy to investigate more personalized therapies against cancers. In addition, SIRT3 expression levels affect sensitivity to chemotherapeutic agents in HCC [26].

In this study, we aimed to investigate the correlation between SIRT3 expression and glucose metabolism and proliferation in HCC. In addition, because a few compounds have been explored to modulate SIRT3 activity [27, 28], we also attempted to identify effective compounds that increase the endogenous SIRT3 modulation mediated by the anti-cancer effect of sorafenib.

## Methods

### Human HCC samples

This study was approved by the Institutional Review Board at Yonsei University Health System Severance Hospital (Seoul, South Korea), and the study was conducted using the current guidelines for ethical research (Yonsei IRB number: 4–2015-0904). The selection of patients was performed as described previously [29].

### Chemicals

PD0332991 was purchased from TOCRIS Bioscience (Bristol, UK) and sorafenib was purchased from Santa Cruz (Dallas, TX, USA). PD0332991 and sorafenib were dissolved in DMSO (Sigma Aldrich, St. Louis, MO, USA) at a concentration of 10 mM. All reagents were stored at − 80 °C.

### Cell lines and cell culture

The human HCC cell lines HepG2, Hep3B, skHep1, and Huh7 were purchased from the Korean Cell Line Bank. HepG2 was cultured in RPMI, and Hep3B, skHep1, and Huh7 were cultured in Dulbecco’s modified Eagle’s medium (DMEM). All media were supplemented with 10% fetal bovine serum (FBS; Hyclone) and 1% penicillin streptomycin. Cells were maintained in a humidified incubator with 5% CO_2_ at 37 °C. For the formation of three-dimensional spheroids, Costar® Ultra-Low attachment multiple-well plates (MerkKGaA → Corning, Darmstadt, Germany) were used. HCC cells were plated at 5000 cells/well and centrifuged at 179×g for 1 min. Spheroids were observed 1–2 days after plating. Hep3B, skHep1, and Huh7 cell lines were plated and incubated for 24 h before transfection. Lipofectamine or RNAiMAX reagent (Invitrogen, Carlsbad, CA, USA) was used to perform siRNA transfection following the manufacturer’s instructions. The plasmids for hSIRT3 (sc-61,555-SH) or scramble shRNA (sc-108,060) were cotransfected into HepG2 cells using Lipofectamine 2000 (Invitrogen, 12,566,014). After 72 h of incubation, the cells were treated with puromycin (2 μg/mL) to generate stable cell line clones.

### Cell proliferation assay and glucose measurement

WST-1 colorimetric assays (Roche, Mannheim, Germany) for cell viability were performed 48 h after treatment according to the manufacturer’s recommendations. Huh7 cells were placed in 96-well plates and being transfected with MOCK or pcDNA-SIRT3 plasmid. After 48 h of treatment, the glucose uptake was determined using Glucose Assay (Promega, Germany) according to the manufacturer’s recommendation. Absorbances at 440 nm and 640 nm were measured using a microplate reader (Molecular Devices, CA, USA).

### RNA isolation and sequencing

Total RNA was isolated using TRIzol reagent (Invitrogen). RNA quality was assessed by Agilent 2100 bioanalyzer using the RNA 6000 Nano Chip (Agilent Technologies, Amstelveen, The Netherlands), and RNA quantification was performed using ND-2000 Spectrophotometer (Thermo Inc., DE, USA). For control and test RNA samples, library was constructed using QuantSeq 3′ mRNA-Seq Library Prep Kit (Lexogen, Inc., Austria) according to the manufacturer’s instructions. Briefly, 500 ng total RNA was prepared for each sample, an oligo-dT primer containing an Illumina-compatible sequence at its 5′ end was hybridized to the RNA, and reverse transcription was performed. After degradation of the RNA template, second strand synthesis was initiated by a random primer containing an Illumina-compatible linker sequence at its 5′ end. The double-stranded library was purified using magnetic beads to remove all reaction components. The library was amplified to add the complete adapter sequences required for cluster generation. The amplified library was purified, and high-throughput sequencing was performed as single-end 75 sequencing using NextSeq 500 (Illumina, Inc., USA).

### Real-time PCR

Total RNA was extracted with TRIzol (Invitrogen) and cDNA was synthesized from 500 ng of total RNA using the ReverTra Ace qPCR RT Master Mix with gDNA Remover (Toyobo, Osaka, Japan). Quantitative RT-PCR was conducted on C1000 a→ a C1000 Thermal Cycler (Bio-Rad) using SYBR Green Real-time PCR Master Mix (Toyobo, Osaka, Japan). Gene expression levels were normalized with beta-2 microglobulin (B2M) mRNA expression levels of corresponding cDNA samples. All PCR primers were purchased from Bioneer (Daejeon, Korea). The following primers were used: SIRT3 (Forward 5′-GAAACTACAAGCCCAACGTCA-3′, Reverse 5′-AAGGTTCCATGAGCTTCAACC-3′), RB1 (Forward 5′-GAAGCAACCCTCCTAAACCAC-3′, Reverse 5′-CTGCTTTTGCATTCGTGTTCG-3′), and B2M (Forward 5′-TTACTCACGTCATCCAGCAGA-3′, Reverse 5′-AGAAAGACCAGTCCTTGCTGA-3′).

### Western blotting

Western blotting was performed as described previously [29]. The primary antibodies in the present study were: SIRT3 (Cell Signaling Technology, Danvers MA, USA; clone C73E3; dilution 1:1000), CDK4 (DCS156, 1:1000), CDK6 (DCS83, 1:1000), Phospho-Rb (Ser807/811) (D20B12, 1:1000), Rb (4H1, 1:2000), PCNA (D3H8P, 1:2000), GLUT1 (1:2000) from Abcam (Cambridge, UK), and Ki67 (Santa Cruz, Dallas TX, USA; MIB-1, 1:500). Western blotting experiments from biological replicates showed similar expression data, attesting to the reproducibility of the results. We used ChemiDoc XRS (Biorad), which enables direct digital visualization of chemiluminescent western blots for the image of signals accumulated in the chemiluminescence reaction. For band quantification, images were analyzed using Image Lab software (Bio-Rad, Hercules, California, USA).

### Flow cytometry analysis

For quantification of apoptosis, double staining was performed according to the manufacturer’s instructions using Annexin V-FITC Apoptosis Detection Kit (BD Pharmingen™, NJ, USA) and propidium iodide (PI). After HepG2 and Huh7 cells were collected after incubation with indicated compound, cells were washed twice with ice-cold PBS and resuspended in 200 μL of binding buffer. Annexin V-FITC was added to the cells and incubated for 15 min in the dark at 25 °C. PI (10 mL) was added to the tube followed by 5 min of incubation at 4 °C in the dark. After incubation, the samples were analyzed by a flow cytometer using CELL Quest software (BD) and 1.0 × 10^5^ events per sample were counted. The fraction of cell population in different quadrants was analyzed using quadrant statistics. Cells in the lower right quadrant (Annexin-V+/PI−) represented early apoptosis and those in the upper right quadrant (Annexin-V+/PI+) represented late apoptosis. For cell cycle analysis, after HepG2 and Huh7 cells were collected after incubation with indicated compound, the cells were incubated in 70% ethanol at 4 °C for 1 h. After washing with PBS, cells were incubated with PI at a concentration of 5 μg/mL and RNaseA at a concentration of 10 mg/mL for 30 min–4 h at 37 °C. The DNA contents were analyzed using FlowJo Software (Tree StarInc., Ashland, OR, USA).

### Migration assay

Chemomigration assays were performed using 24-well plates with uncoated polycarbonate membrane inserts (BioCoat; BD Biosciences, Heidelberg, Germany). A total of 50,000 cells in medium containing 0.1% FBS and sorafenib, PD0332991, or combination of sorafenib and PD033291 were added onto the insert. The lower well was filled with a medium supplemented with 10% FBS. Twenty-four hours later, the cells that had migrated were fixed in 100% methanol and stained with 1.5% (w/v) toluidine blue in water. Images were recorded using an Olympus BX53 microscope with Olympus Cell Sens software (Carl Zeiss Microscopy, GmbH, Jena, Germany).

### Immunostaining

Immunohistochemistry (IHC) and immunofluorescence (IF) were performed as described previously [30]. After antigen retrieval, immunostaining was performed using various antibodies. The primary antibodies used were: SIRT3 (Cell Signaling Technology, Danvers MA, USA; clone C73E3; dilution 1:500); Ki67 (Dako, Glostrup, Denmark; MIB-1; 1:500); GLUT1 (1:500), and Ki67 (SP6, 1:500) from Abcam (Cambridge, UK). Images were recorded using an Olympus BX53 microscope with Olympus Cell Sens software (Carl Zeiss Microscopy, GmbH, Jena, Germany). The percentage of Ki67-positive cells and phosphorylated retinoblastoma protein (pRb) was calculated by counting the number of cells with DAPI-stained nuclei.

### The Cancer genome atlas (TCGA) data analysis

mRNA levels of TCGA liver HCC data were obtained from the OncoLnc TCGA data portal (www.oncolnc.org). A set of 360 HCC samples with high and low gene expression groups (50–50 percentile) was used for correlation graphs of two different genes. GraphPad Prism 5 (GraphPad Software, San Diego, CA, USA) was used for mapping.

### Statistical analysis

Statistical analyses were performed using GraphPad Prism Software (GraphPad Software, Inc., San Diego, CA). Results are expressed as mean ± SE (range). *P* values < 0.05 were considered statistically significant. Comparisons between groups were made using the Mann-Whitney test.

## Results

### Expression of SIRT3 in patients with HCC

**Fig. 1:**
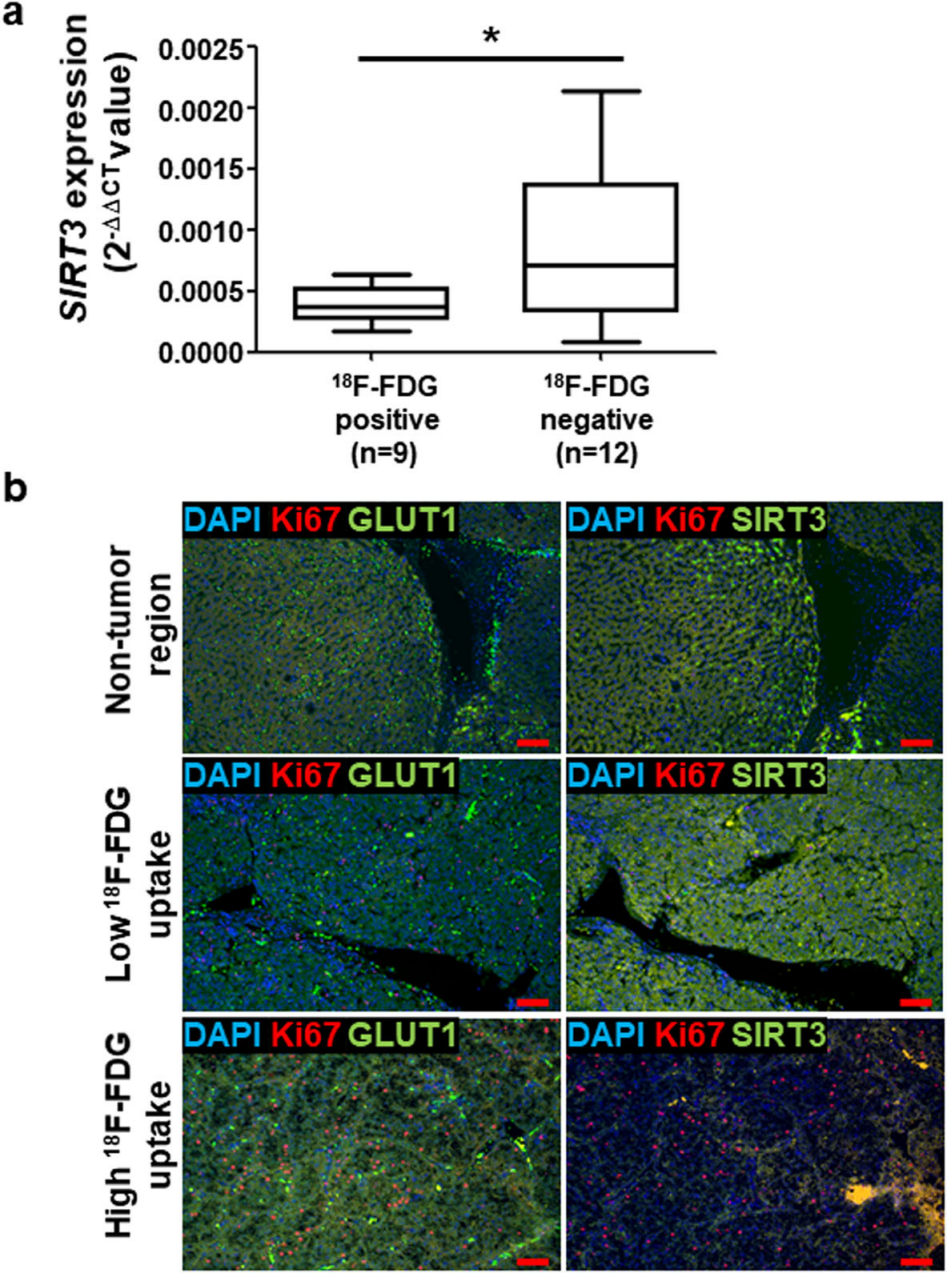
SIRT3 expression in patients with hepatocellular carcinoma (HCC) and with different 18F-FDG uptake. **a.** RNA was extracted from frozen HCC samples obtained after transsphenoidal surgery. RT-PCR was performed using probe sets specific for *SIRT3*. The expression of the target genes was normalized to that of *B2M* (housekeeping gene) using the 2^−ΔΔCt^ method. The boundary of the box closest to zero indicates the 25th percentile, the line within the box marks the median, and the boundary of the box farthest from zero indicates the 75th percentile. **b.** Formalin-fixed, paraffin-embedded human HCC samples were used and immunofluorescence was performed using the indicated antibodies and counterstained with DAPI. Scale bars: 50 μm. Statistical analyses were performed using GraphPad Prism. Results are expressed as mean ± SD. Comparisons between groups were made using the Mann-Whitney test. *P < 0.05

Imaging with 18F-fluorodeoxyglucose (FDG) positron emission tomography/computed tomography (PET/CT) was used to evaluate glucose metabolism. To investigate the correlation between glucose uptake and SIRT3 expression, 21 patients with HCC were divided into two groups according to 18F-FDG uptake: 9 patients with high glycolytic HCC with high 18F-FDG uptake and 12 with low glycolytic HCC with low 18F-FDG uptake. The mRNA expression of *SIRT3* was higher in the low glycolytic group than in the high glycolytic group (Fig. 1a). To confirm our observation, we performed IHC analysis with HCC tissues from the two groups (*n* = 6 in each group). In patients with low FDG uptake, low membranous GLUT1 expression, low Ki67 expression, and high SIRT3 expression were observed in the tumor region (Fig. 1b and supplementary data 1). High Ki67 and low SIRT3 expression levels were observed in patients with high GLUT1 expression in the high FDG uptake group. In addition, we also confirmed the expression of SIRT3 in patients with HCC by western blotting (supplementary data 2). Downregulation of SIRT3 was determined also in HCC patients with high 18F-FDG uptake compare with low 18F-FDG uptake. Altogether, SIRT3 expression seemed to be associated with glycolytic metabolism and cell proliferation in patients with HCC.

### Differential expression of SIRT3 in HCC cells

**Fig. 2:**
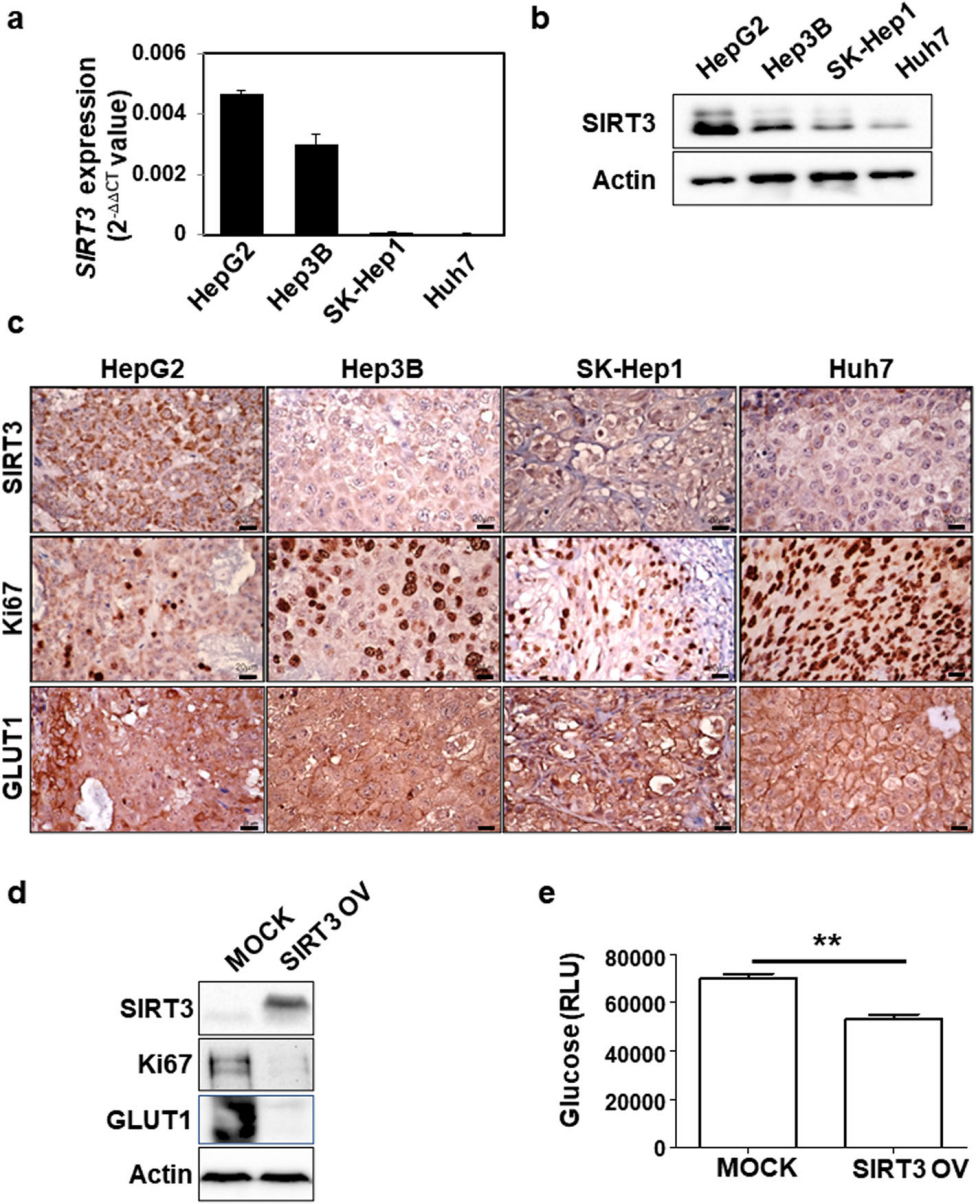
Expression of SIRT3 in hepatocellular carcinoma (HCC) cell lines and HCC xenograft models. **a***SIRT3* expression in four different HCC cell lines was measured using quantitative RT-PCR. The expression level of target genes was normalized to that of the housekeeping gene *B2M* using the 2^−ΔΔCt^ method. Data are shown as the mean of three independent experiments ± SD. **b** Western blotting in different HCC cell lines using antibodies against SIRT3 and actin. The images shown here are cropped and the full-length blots/gels are presented in Additional file 2: Fig. S1. **c** Formalin-fixed, paraffin-embedded liver tissues from HCC xenograft model were used. Immunohistochemistry was performed using antibodies against SIRT3, GLUT1, and Ki67 and counterstained with hematoxylin. Scale bars: 20 μm. **d** Huh7 cells were transfected with MOCK vector and pcDNA-SIRT3. After 48 h of incubation, protein was extracted and the expression of SIRT3, Ki67, and actin was determined using western blotting. The images shown here are cropped and the full-length blots/gels are presented in Additional file 2: Fig. S2. **e** Glucose uptake was measured using Glucose-Glo Assay. Data are shown as the mean of three independent experiments ± SD. Statistical analyses were performed using GraphPad PrismComparisons between groups were made using the Mann-Whitney test. *P < 0.01

The expression of SIRT3 was assessed in three HCC cell lines (HepG2, Hep3B and Huh7), liver adenocarcinoma cell line with characteristics of liver sinusoidal endothelial cells (SK-Hep1) [31], and their xenograft models with different rates of proliferation and glycolysis. HepG2 cells showed the highest expression of SIRT3 at the mRNA and protein levels (Fig. 2a, b and supporting data),.. Similar to human patients with HCC, an inverse correlation between GLUT1 and SIRT3 was observed in an HCC xenograft model (Fig. 2c). Moreover, there was a significant negative correlation between SIRT3 and Ki67. HepG2 and Hep3B cells had low Ki67 and high SIRT3 expression, whereas Huh7 and SK-HEP1 cells had high Ki67 and low SIRT3 expression. These results were consistent with the TCGA database analysis of human HCC samples (Supporting data 3A and 1B). Spearman’s correlation also showed a significant negative correlation between *SIRT3* and *Ki67* (Spearman’s coefficient r = − 0.3093, *P* < 0.0001), and *SIRT3* and *HK2* (Spearman r = − 0.239, *P* < 0.0001). To further corroborate our findings, SIRT3 was overexpressed in Huh 7 cells with low basal SIRT3 expression. Overexpression of SIRT3 reduced the expression of Ki67 and GLUT1 and also significantly reduced glucose uptake (Fig. 2d and e). Taken together, our results indicate that SIRT3 expression is negatively correlated with glycolytic metabolism and proliferation in HCC cells and in their xenograft models.

### The relationship between SIRT3 expression levels and sorafenib treatment

**Fig. 3:**
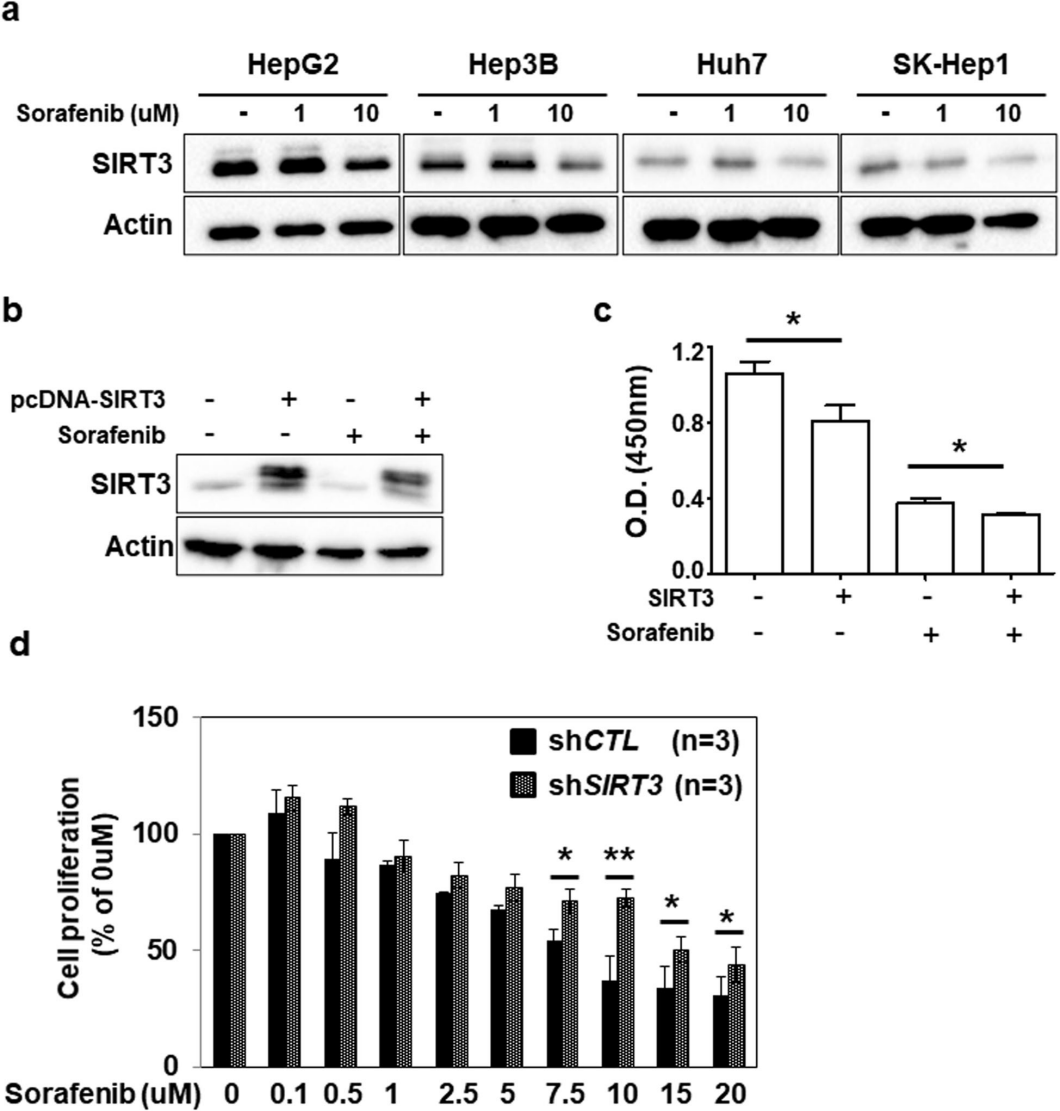
The relationship between SIRT3 expression levels and sorafenib treatment. **a** Reduced SIRT3 expression upon sorafenib treatment in hepatocellular carcinoma (HCC) cells. HCC cells were incubated with DMSO, 1 μM sorafenib, or 10 μM sorafenib. After 48 h, the level of SIRT3 and actin was measured by western blotting. The images shown here are cropped and the full-length blots/gels are in Additional file 2: Fig. S3. **b** Huh7 cells were transfected with MOCK or pcDNA-SIRT3. After 24 h, the cells were treated with 10 μM sorafenib for further 24 h and then western blotting was carried out to determine SIRT3 and β-actin expression. The images shown here are cropped and the full-length blots/gels are in Additional file 2: Fig. S4. **c** Cell proliferation was measured using WST-1 assays. Data are shown as the mean of three independent experiments ± SD. **d** SIRT3 KD or control stable cell line were incubated with indicated concentration of sorafenib. After 48 h, cell proliferation was measured using WST-1 assays. Statistical analyses were performed using GraphPad Prism. Results are expressed as mean ± SD. Comparisons between groups were made using the Mann-Whitney test. *P < 0.05; **P < 0.01

Because our results suggested a crucial role of SIRT3 in HCC, we next investigated whether the expression of SIRT3 was changed by sorafenib treatment by incubating HCC cell lines with sorafenib for 48 h. We observed that the expression of SIRT3 in HCC cells decreased after treatment with 10 μM sorafenib (Fig. 3a). Furthermore, a more prominent decrease in Huh7 cell proliferation was observed in cells transfected with SIRT3 than in cells transfected with MOCK (Fig. 3b and c). In addition, we generated SIRT3 KD stable cell line using HepG2 cells. The sensitivity against sorafenib was significantly reduced in SIRT3 KD stable cells compare with control stable cells, suggesting that the expression of SIRT3 enhances the sensitivity of HCC cells to sorafenib (Fig. 3d and supplementary data 4). Together, these results suggest that modulation of SIRT3 might be an effective strategy to increase the sensitivity to sorafenib.

### Negative correlation between SIRT3 and pRb

**Fig. 4:**
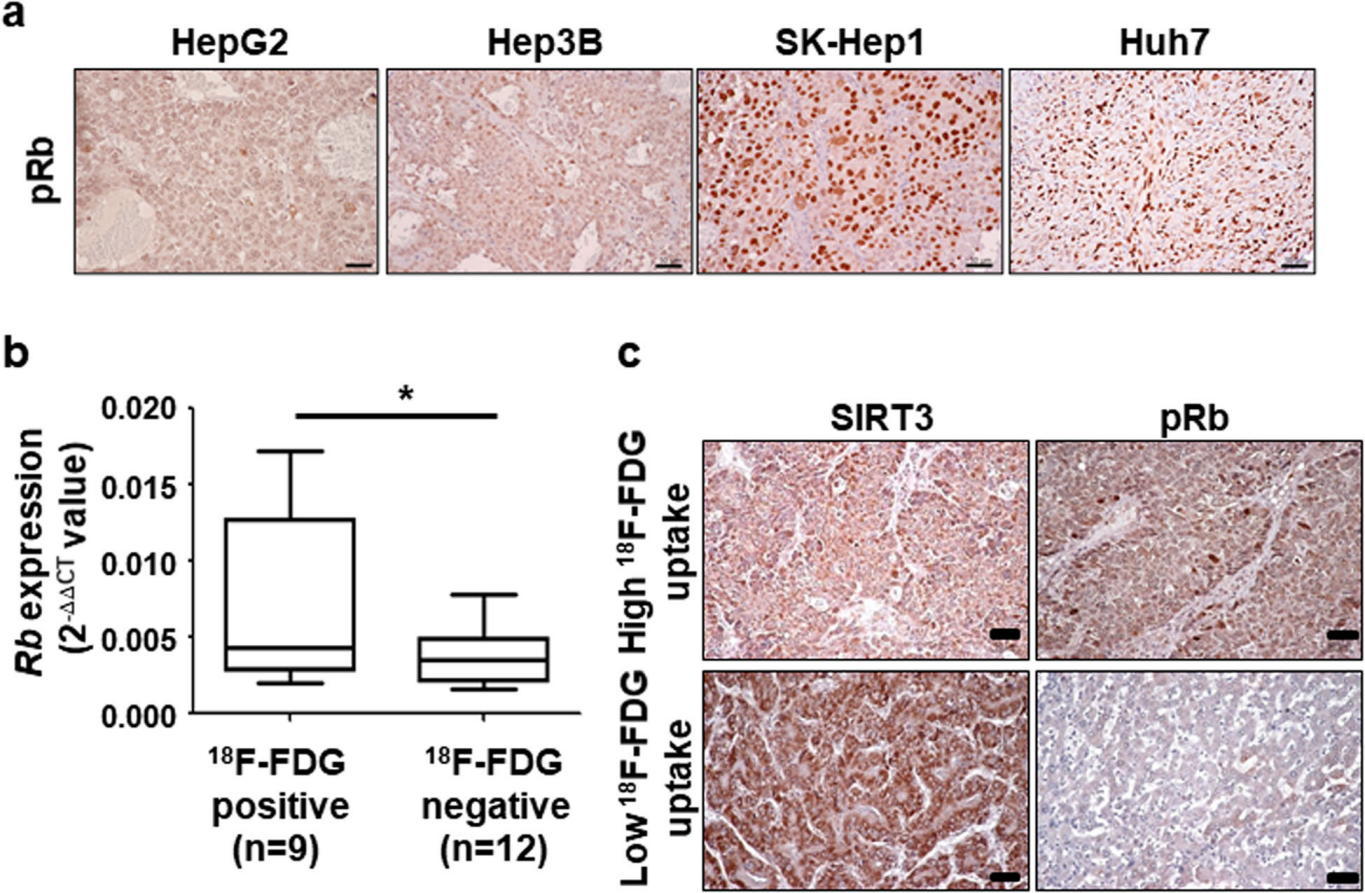
Negative correlation between SIRT3 and retinoblastoma protein (Rb). **a** Formalin-fixed, paraffin-embedded (FFPE) liver tissues from HCC xenograft model were used. Immunohistochemistry was performed using antidies against phosphor-Rb (pRb) and counterstained with hematoxylin. Scale bars: 50 μm. **b** RNA was extracted from frozen HCC samples obtained after transsphenoidal surgery. RT-PCR was performed using probe sets specific for *Rb*. The expression of the target genes was normalized to that of *B2M* (housekeeping gene) using the 2^−ΔΔCt^ method. The boundary of the box closest to zero indicates the 25th percentile, the line within the box marks the median, and the boundary of the box farthest from zero indicates the 75th percentile. *P < 0.05. **c** FFPE liver tissues from human patients with HCC were used. Immunohistochemistry was performed using antibodies against SIRT3 (left) and pRb (right) and counterstained with hematoxylin. Scale bars: 50 μm

PD0332991, a highly selective inhibitor of CDK4/CDK6 kinases with the ability to block phosphorylation activity of Rb, has become a novel therapeutic candidate for HCC [32]. We assessed the expression of pRb in patients with HCC to determine whether PD0332991 can be a candidate drug to increase the sensitivity of sorafenib by modulation of SIRT3 expression. IHC was performed using antibody specific for pRb using HCC xenograft models. Huh7 and skHep1-xenograft models with low SIRT3 expression showed high expression of pRb. Thus, the expression of pRb and SIRT3 had a negative correlation in HCC xenograft models (Fig. 4a and supplementary data 5A). In human HCC, Rb mRNA levels were significantly higher and those of SIRT3 mRNA were relatively lower in the high 18F-FDG group than in the low 18F-FDG group (Fig. 4b). Spearman’s correlation also showed a significant negative correlation between *SIRT3* and *Rb1* (Spearman’s coefficient r = − 0.3408, *P* < 0.0001) expression levels in human HCC patients by TCGA data analysis (Supplementary data 3D). Moreover, we also observed a negative correlation between the expression of pRb and SIRT3 in human patients with HCC (Fig. 4c and Supplementary data 5B).

### SIRT3 expression is upregulated upon treatment with a CDK4/6 inhibitor

**Fig. 5:**
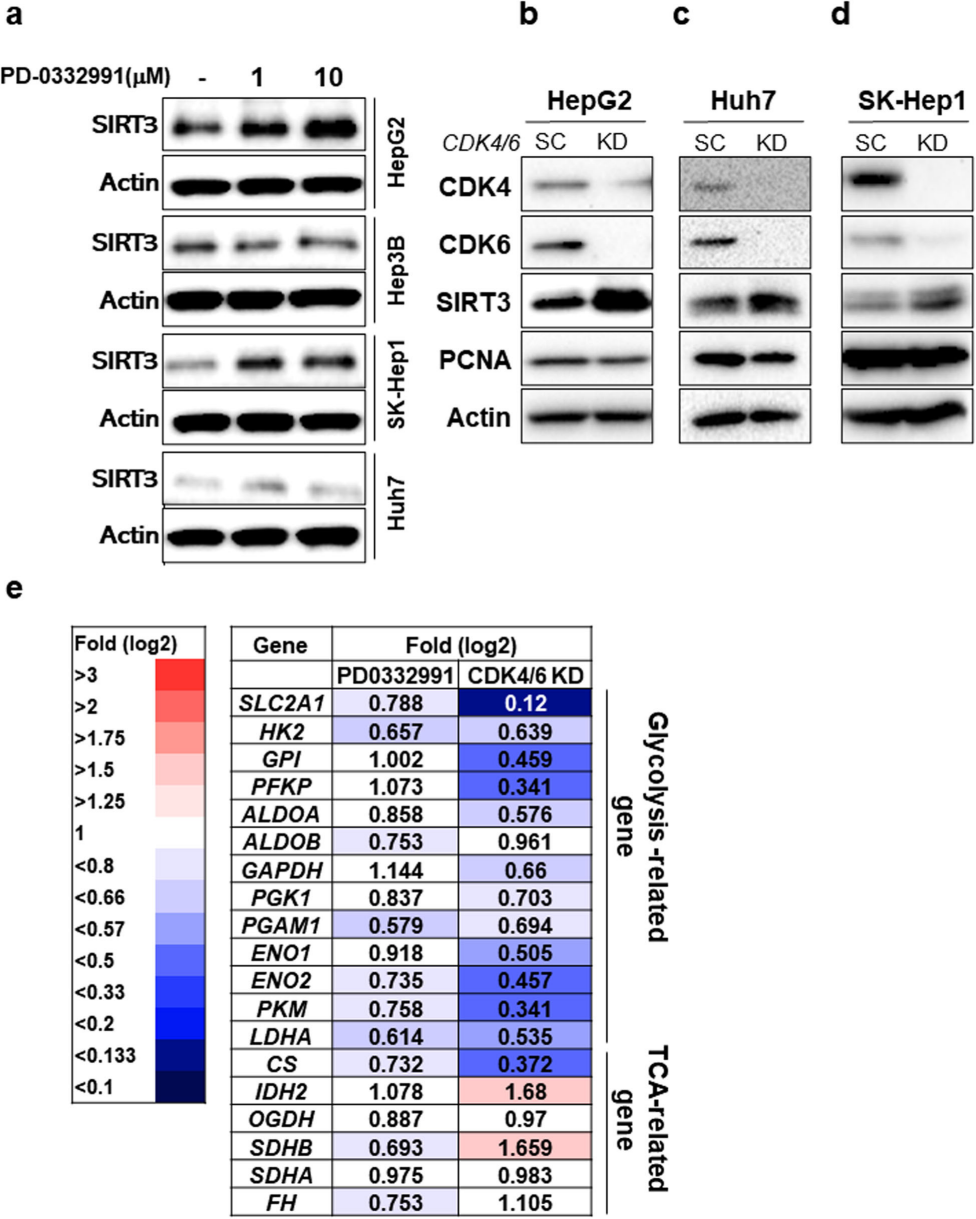
SIRT3 induction after PD0332991 treatment. **a** HepG2, Hep3B, SK-Hep1, and Huh7 cells were incubated with DMSO, 1 μM PD0332991, or 10 μM PD0332991. After 48 h, SIRT3 and actin levels were evaluated using western blotting. The images shown here are cropped and the full-length blots/gels are presented in Additional file 2: Fig. S5. **b-d** HepG2 cells (b), Huh7 cells (c), SK-Hep1 cells (d) were transfected with scrambled siRNA oligos or siRNA oligos against *CDK4/6*. After 48 h, western blotting was performed to detect indicated proteins. The images shown here are cropped and the full-length original blots are shown in Additional file 2: Fig. S6, S7 and S8. **e** Fold change of the indicated genes obtained by gene expression profiling of HepG2 cells after transfection with scrambled siRNA oligos or siRNA oligos targeting *CDK4/6*. All data are uploaded into the GEO database under the accession number GSE145389

Because a connection between SIRT3 and pRb was identified in this study, we investigated whether PD0332991 can modulate the expression of SIRT3 to enhance the sorafenib sensitivity in HCC cells. Upon PD0332991 treatment, the expression of SIRT3 increased in HepG2. In SK-HEP1 and Huh7 cells, SIRT3 expression increased marginally compared with that in the control (Fig. 5a). However, no significant change was observed in Hep3B cells. It is possible that Hep3B cells present mutations of Rb that underlie the relative resistance to CDK4/6 inhibition [33, 34]. Therefore, Hep3B cells were excluded from further experiments. In addition to PD0332991 treatment, we investigated the expression of SIRT3 upon knockdown of *CDK4/6* in HCC cells. Similar to that after PD0332991 treatment, SIRT3 expression was upregulated in CDK4/6 knockdown HepG2 cells, Huh7 cells and SK-Hep1 cells (Fig. 5b-d). The expression of PCNA, a proliferation marker, decreased upon *CDK4/6* silencing, which had an effect similar to that of treatment with PD0332991 (Fig. 5b-d).

Our data suggested that SIRT3 expression is negatively correlated with glucose metabolism (Fig. 1a and b). Thus, we assessed the expression of glycolysis- and TCA-related genes after CDK4/6 inhibition in HepG2 cells by microarray analysis. A reduction in the expression of glycolysis-related genes, including *SLC2A1* (fold change: 0.12), *PFKP* (fold change: 0.341), *PKM* (fold change: 0.457), and *HK2* (fold change: 0.693) was observed in CDK4/6 KD HepG2 cells (Fig. 5e). In addition, the most dysregulated genes in the two sample groups (scramble vs. *CDK4/6* KD) were associated with the following categories: DNA replication, meiotic cell cycle process, chromosome segregation, regulation of fatty acid oxidation, lipid catabolic process, and regulation of lipid catabolic process (Supporting data 3). The rate of dysregulation in glycolysis-related genes after PD0332991 treatment was smaller compared with that after CDK4/6 KD (Fig. 5e). Thus, we identified a novel mechanism to modulate SIRT3 expression by CDK4/6 inhibition, resulting in the inhibition of glycolysis and cell proliferation.

### Enhancement of anti-cancer effect of sorafenib during combination treatment with PD0332991

**Fig. 6:**
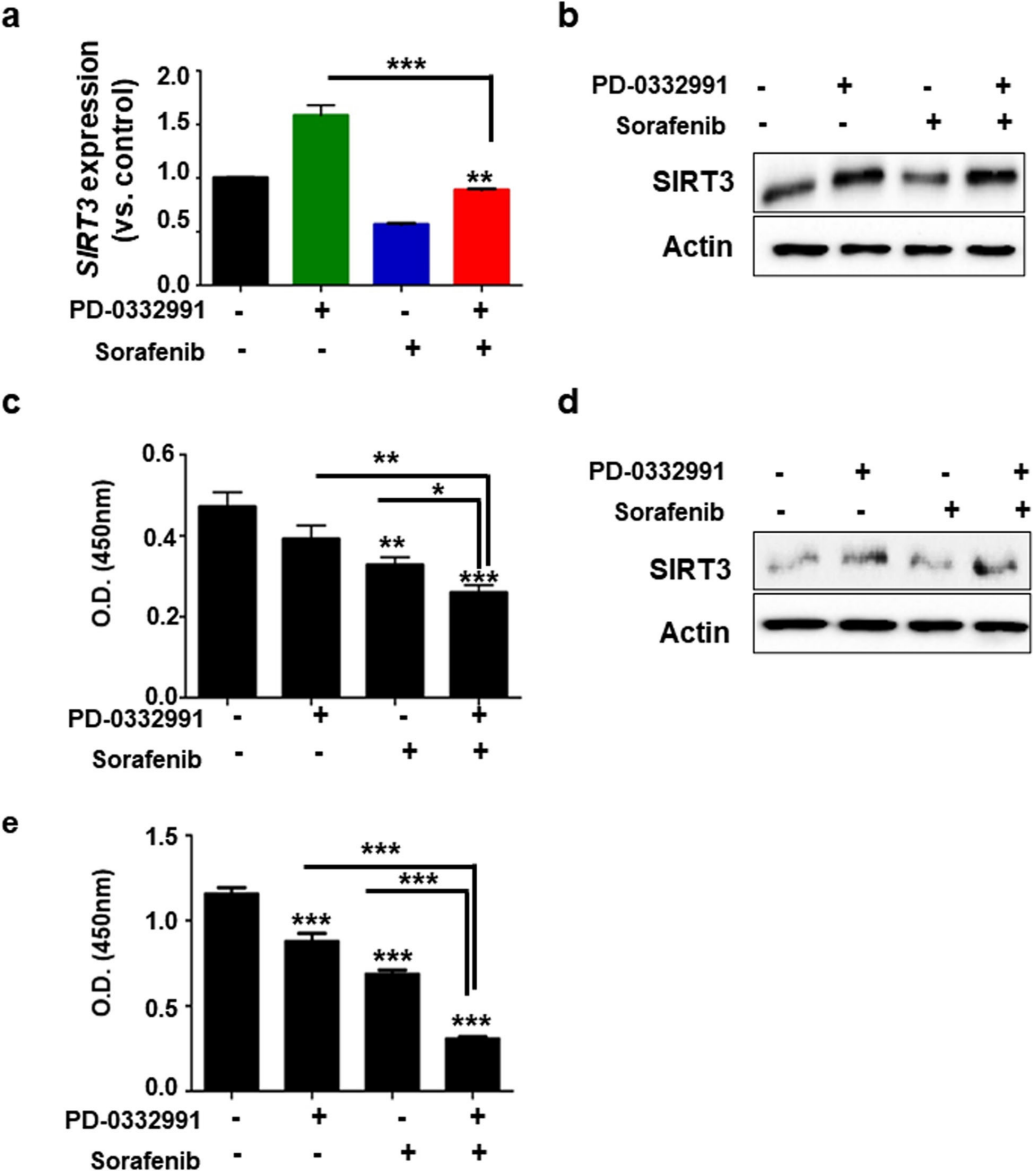
Increased sensitivity to sorafenib after combined treatment with sorafenib and PD0332991. **a** HepG2 cells were incubated with DMSO or 10 μM PD0332991 with or without 1 μM sorafenib. After 48 h, *SIRT3* levels were analyzed using qRT-PCR. The expression level of target genes was normalized to that of the housekeeping gene *B2M* using the 2^−ΔΔCt^ method. Data are shown as the mean of three independent experiments ± SD. **b** Cells were treated as in (A). Protein was extracted and the expression of SIRT3 and actin was determined using western blotting. The images shown here are cropped and the full-length blots/gels are presented in Additional file 2: Fig. S9. **c** In parallel, proliferation was assessed 48 h later using WST-1 assays. **d** Huh7 cells were incubated with DMSO, or 10 μM PD0332991 with or without 1 μM sorafenib. After 48 h, protein was extracted and the expression of SIRT3 and actin was determined using western blotting. The images shown here are cropped and the full-length blots/gels are presented in Additional file 2: Fig. S10. **e** In parallel, proliferation was assessed 48 h later using WST-1 assays. Data from six technical replicates were analyzed and expressed as the mean ± SD

**Fig. 7:**
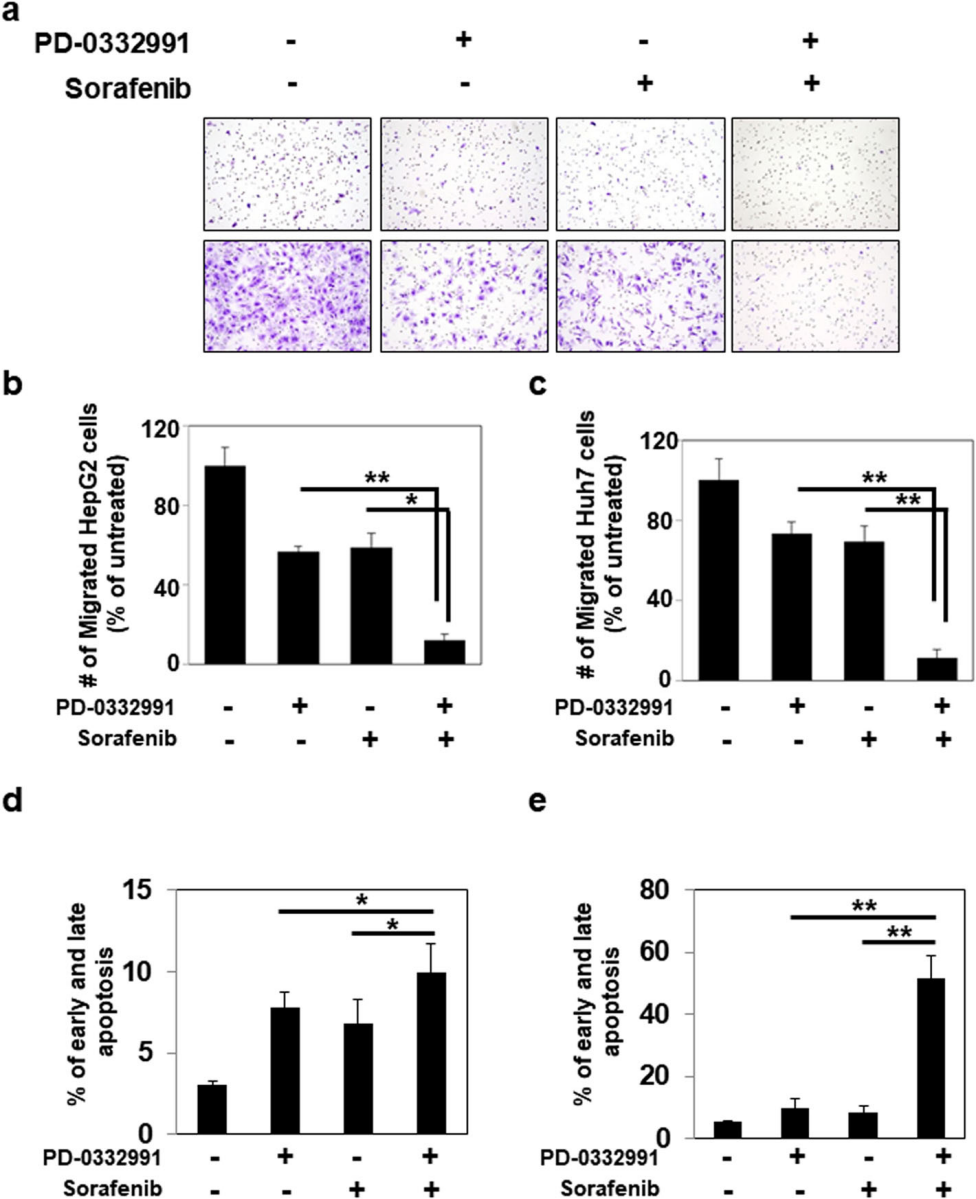
The enhancement of the growth inhibition after combined treatment of sorafenib and PD0332991 in HepG2 cells and Huh7 cells. **a** Migration assay was performed. Penetrating HepG2 cells and Huh7 cells after treatment with indicated compound for 24 h were fixed and visualized by the staining of crystal violet. **b, c** Quantitative analyses were performed for the cells migrating through the matrigel-coated filter. Five random fields of each test at × 200 magnifications were counted. **d, f** Analysis of apoptosis by Annexin V-APC/propidium iodide (PI) double staining of HepG2 and Huh7 cells after 24 h treatment with indicated compound. Two-color flow cytometry dot plots show the percentages of living cells as negative for both annexin V and PI; early-stage apoptotic cells as the populations testing Annexin V positive and PI negative, and late-stage apoptotic/necrotic cells as double-positive cells. Results are represented in as mean ± SD, *n* = 3. Statistical analyses were performed using GraphPad Prism. Results are expressed as mean ± SE (range). Comparisons between groups were made using the Mann-Whitney test.*, P < 0.05;**, *P* < 0.01;;***, *P* < 0.001

We next aimed to investigate whether upregulation of SIRT3 by the CDK4/6 inhibitor PD0332991 could enhance the anti-cancer effect of sorafenib on HCC cells. We performed combination treatment with sorafenib and PD0332991 in HepG2. Both SIRT3 mRNA and protein expression were upregulated in HepG2 cells exposed to the two drugs (Fig. 6a and b). In these conditions, we also noticed a more pronounced reduction of cell viability compared with single treatment (Fig. 6c). To confirm, we generated 3-dimensional spheroids from HepG2 cells. The spheroid size of the HepG2 cells after the combined treatment was more reduced, compared with that after single treatment. We also performed western blotting and observed increased level of SIRT3 after PD0332991 treatment, and after the combined treatment, compared with that after control and sorafenib treatment (Sup data 7). Huh7 cells showed the same results as HepG2 with the exception of SIRT3 mRNA expression because of the very low basal level of SIRT3 mRNA in Huh7 cells (Fig. 6d and e). To confirm the increased anti-cancer effect of the combined treatment, we also measured migration of cells after treatment with soranfenib, PD0332991 or their combination. The migration of HepG2 and Huh7 cells were further reduced after the combined treatment compared with that after single treatment (Fig. 7a, b and c). In parallel, we performed cell cycle analysis, immunostaining of ki67 and apoptosis assay in HepG2 cells and Huh7 cells. In cell cycle analysis, we observed reduced reduction of G2/M phase compare with single treatment in HepG2 cells. In Huh7, G2/M and S phase were reduced after combined treatment with both compound (Supplementary data 8A). Also, immunostaining of Ki67 was determined in HepG2 and huh7 cells after combined treatment. Ki67 positive cells were dramatically decreased in cells with combined treatment (Supplementary data 8B and C). Moreover, annexin V-PI staining demonstrated that combined treatment with sorafenib and PD033291 increased early and late apoptosis (Fig. 7d and supplementary data 8D). Altogether, these results show that upregulation of SIRT3 by CDK4/6 inhibition enhances the sensitivity to sorafenib treatment in HCC cells.

## Discussion

Several studies have emphasized the importance of SIRT3 in carcinogenesis [9, 24, 35]. However, there have been few studies on the mechanisms that control SIRT3 expression or on the discovery of clinically applicable drugs that can modulate it. In this study, we investigated a novel function of CDK4 /6 inhibitor as an inducer of SIRT3, resulting in enhanced sensitivity to sorafenib treatment in HCC cells.

To date, *SIRT3* is known as a tumor suppressor gene [23], and overexpression of SIRT3 reduces cell growth and proliferation in HCC [23, 25, 26]. SIRT3 also induces apoptosis in abnormal cells through the upregulation of MnSOD, p53, Bax, and Fas [19]. Wang et al., determined patient survival and outcome in patients with HCC according to SIRT3 expression [36]. In fact, reduced expression of SIRT3 was associated with poor prognosis, whereas intra-tumoral SIRT3 expression was reported as a good prognostic factor in the early stages. So far, the effect of SIRT3 on glucose metabolism has been studied in cancers other than HCC [37–39]. Finley et al. proposed that SIRT3 loss increases ROS levels and promotes tumorigenesis by altering global cellular metabolism [24]. In this study, patients with HCC were divided into low and high glycolytic groups by 18F-FDG-PET analysis. Consistent with previous results with other tumor types, we found high SIRT3 and low Ki67 expression in the low glycolytic group of patients with HCC. Thus, our study suggests that SIRT3 expression is associated with glycolysis and proliferation in human HCCs. Indeed, upregulation of SIRT3 by CDK4/6 inhibition and treatment with PD0332991 induced the downregulation of glycolysis-related genes in our gene analysis.

Selective CDK4/6 inhibitors, including PD0332991, are currently used for the treatment of a variety of tumor types, including breast cancer, melanoma, and non-small cell lung cancer [40–42]. In this study, we found that CDK4/6 inhibition by treatment with siCDK4/6 or PD0332991 upregulated SIRT3 expression. Previous studies have determined that SIRT1 is involved in the regulation of SIRT3 expression by deacetylation and binding as a transcription factor [43]. In addition, SIRT1 is involved in the deacetylation of retinoblastoma (Rb), leading to dissociation of E2F1 and enhanced cell proliferation [44]. Therefore, there might be a correlation among SIRT3, SIRT1, and pRB expression levels in HCC cells. Indeed, there was a negative correlation between SIRT1 and SIRT3 expression in data from patients with HCC from the TCGA database (Supplementary Fig. 1C). However, the mechanism of SIRT3 expression by CDK4/6 inhibition remains unclear, and should be investigated in future studies.

Sorafenib has not been effective in patients with advanced HCC, and its use is often associated with reduction of drug sensitivity. Therefore, it is very important to identify a drug candidate that can replace or be used together with sorafenib. Tao et al. found that upregulation of SIRT3 expression can enhance the sensitivity of HCC cells to chemotherapeutic agents [26]. In our study, we found that the upregulation of SIRT3 by transfection in HCC cells reduced cell proliferation and significantly increased sensitivity to sorafenib. Moreover, the restoration of SIRT3 by PD0332991 could increase sensitivity to sorafenib, resulting in enhanced inhibition of proliferation and migration in HCC cells.

Thus, we propose SIRT3 expression as a predictor of sorafenib response. To concrete our observations in the in vitro system, preclinical studies will be conducted in the future.18F-FDG is a surrogate imaging modality to measure glucose metabolism in patients with HCC. However, there are few studies on 18F-FDG imaging in patients treated with sorafenib [45]. Our results prove the negative correlation between the expression of SIRT3 and glucose metabolism using human HCC tissues and HCC cells in vitro. Since the expression of SIRT3 is a predictor of response to sorafenib, [18F] FDG-PET imaging could monitor the drug sensitivity in HCC patients clinically during sorafenib treatment.

## Conclusion

In summmary, our data indicate the importance of CDK4/6 inhibitors as a new approach to improve HCC therapy. Moreover, our study shows that induction of SIRT3 by CDK4/6 inhibition causes inhibition of cell growth and glucose metabolism and increased susceptibility to chemotherapy. Thus, the modulation of SIRT3 might be a novel treatment in patients with HCC and, possibly, other cancers in which SIRT3 acts as a tumor suppressor.

## Supplementary information

**Additional file 1 Supplementary Data 1**. Quantification of immunostaining. (A, C) Mean fluorescent intensity (MFI) indicating the expression of (A) SIRT3 and membranous (C) GLUT1 from 12 patients with high FDG uptake (*n* = 6) and low FDG uptake (n = 6). Quantification of fluorescence in microscopic images stained with GFP (Green) and DsRed (Red) was carried out using IMT i-Solution software (Martin Microscope Company, Easley, USA). (B) Ki67 positive cells in positive and negative tumor regions of indicated proteins in HCC with high FDG uptake. Statistical analyses were performed using GraphPad Prism. Results are expressed as mean ± SD. Comparisons between groups were made using the Mann-Whitney test. *P < 0.05; **P < 0.01. **Supplementary Data 2.** SIRT3 expression in patients with hepatocellular carcinoma (HCC) and with different 18F-FDG uptake. (A) Protein was extracted from frozen HCC samples obtained after transsphenoidal surgery. Western blotting was performed using antibodies against SIRT3 and actin. The images shown here are cropped and the full-length blots/gels are presented in Additional file 2: Fig. S11. (B) Band quantification was carried out using ImageJ. Statistical analyses were performed using GraphPad Prism. Results are expressed as mean ± SE. Comparisons between groups were made using the Mann-Whitney test. *P < 0.05. **Supplementary Data 3.** TCGA data analysis. The indicated mRNA level of the Cancer Genome Atlas (TCGA) Liver Hepatocellular Carcinoma data was obtained from OncoLnc (www.oncolnc.org) TCGA data portal. **Supplementary Data 4.** SIRT3 expression in SIRT3 knockdown and control stable clones. The ratios of the band intensities were normalized by actin and are reported below the respective panels. The images shown here are cropped and the full-length blots/gels are presented in Additional file 2: Fig. S12. **Supplementary Data 5.** Quantification of immunostaining. We quantified the positive area of SIRT3 (A) and membranous GLUT1 (C) from indicated xenograft model in Fig. 2c. (B) We counted the Ki67 positive cells in tumor region of Fig. 2c. (C) We quantified the positive cells of pRb from indicated xenograft model in Fig. 4a. (D) Correlation of SIRT3 and pRb was performed in patients with HCC. Statistical analyses were performed using GraphPad Prism. Results are expressed as mean ± SE (range). Comparisons between groups were made using the Mann-Whitney test. *, *P* < 0.05;**, *P* < 0.01. **Supplementary Data 6.** Gene set enrichment analysis (GSEA). **Supplementary Data 7.** The effect of combined treatment of sorafenib and PD033291 on spheroids from HepG2 cells. (A) HepG2 cells were plated in low-affinity 96-well plates. After the formation of spheroids, HepG2 cells were treated with vehicle, 10 μM sorafenib, or PD033291 for the indicated incubation time. (B) Spheroid size was measured using Image J software after microscopy-based imaging based on six individual spheroids in each condition. Data are shown as the mean of three independent experiments ± SD. Data were analyzed using an unpaired t-test. *P < 0.05, **P < 0.01. (C) In parallel, western blotting was performed to detect SIRT3 and actin. The images shown here are cropped and the full-length blots/gels are presented in Additional file 2: Fig. S13. **Supplementary Data 8.** Synergistic antitumoral effects of sorafenib and PD0332991 (A) The cell cycle analysis after treatment with the combination of sorafenib and PD0332991 in HepG2 and Huh7 cells. (B) HepG2 and Huh7 were plated on coverslips in 24-well plates. The next day, the cells were incubated with indicated compound. After 24 h, cells were fixed and processed for immunofluorescent staining with Ki67 protein and counterstained with DAPI. (C) In parallel, we counted Ki67 positive cells. (D) Analysis of apoptosis by Annexin V-APC/propidium iodide (PI) double staining of HepG2 and Huh7 cells. Two-color flow cytometry dot plots show the percentages of living cells as negative for both annexin V and PI; early-stage apoptotic cells as the populations testing Annexin V positive and PI negative, and late-stage apoptotic/necrotic cells as double-positive cells. **Supplementary Data 9.** Quantification of western blotting of Figures.

**Additional file 2 Figure S1.** Full-length original blots of Fig. 2b. **Figure S2.** Full-length original blots of Fig. 2d. **Figure S3.** Full-length original blots of Fig. 3a. **Figure S4.** Full-length original blots of Fig. 3b. **Figure S5.** Full-length original blots of Fig. 5a. **Figure S6.** Full-length original blots of Fig. 5b (HepG2). **Figure S7.** Full-length original blots of Fig. 5c(Huh7). **Figure S8.** Full-length original blots of Fig. 5d (SK-Hep1). **Figure S9.** Full-length original blots of Fig. 6b. **Figure S10.** Full-length original blots of Fig. 6d. **Figure S11**. Full-length original blots of Supporting data 2. **Figure S12.** Full-length original blots of Supporting data 4. **Figure S13.** Full-length original blots of Supporting data 7.

## Data Availability

The datasets used and/or analyzed during the present study are available from the corresponding author on reasonable request.

## Abbreviations

HCC: Hepatocellular carcinoma
RT-PCR: Real-time polymerase chain reaction
FDG: 18F-fluorodeoxyglucose
PET/CT: Positron emission tomography/computed tomography
TCGA: The Cancer Genome Atlas
CDK: Cyclin-dependent kinases
