# Supplementary material for: Modulation of SIRT3 expression through CDK4/6 enhances the anti-cancer effect of sorafenib in hepatocellular carcinoma cells
Source: BMC Cancer. 2020 Apr 19;20:332. doi: 10.1186/s12885-020-06822-4 (PMC7168998; doi:10.1186/s12885-020-06822-4)
Supplement: Supplementary file 1 — Additional file 1 Supplementary Data 1. Quantification of immunostaining. (A, C) Mean fluorescent intensity (MFI) indicating the expression of (A) SIRT3 and membranous (C) GLUT1 from 12 patients with high FDG uptake (n = 6) and low FDG uptake (n = 6). Quantification of fluorescence in microscopic images stained with GFP (Green) and DsRed (Red) was carried out using IMT i-Solution software (Martin Microscope Company, Easley, USA). (B) Ki67 positive cells in positive and negative tumor regions of indicated proteins in HCC with high FDG uptake. Statistical analyses were performed using GraphPad Prism. Results are expressed as mean ± SD. Comparisons between groups were made using the Mann-Whitney test. *P < 0.05; **P < 0.01. Supplementary Data 2. SIRT3 expression in patients with hepatocellular carcinoma (HCC) and with different 18F-FDG uptake. (A) Protein was extracted from frozen HCC samples obtained after transsphenoidal surgery. Western blotting was performed using antibodies against SIRT3 and actin. The images shown here are cropped and the full-length blots/gels are presented in Additional file 2: Fig. S11. (B) Band quantification was carried out using ImageJ. Statistical analyses were performed using GraphPad Prism. Results are expressed as mean ± SE. Comparisons between groups were made using the Mann-Whitney test. *P < 0.05. Supplementary Data 3. TCGA data analysis. The indicated mRNA level of the Cancer Genome Atlas (TCGA) Liver Hepatocellular Carcinoma data was obtained from OncoLnc (www.oncolnc.org) TCGA data portal. Supplementary Data 4. SIRT3 expression in SIRT3 knockdown and control stable clones. The ratios of the band intensities were normalized by actin and are reported below the respective panels. The images shown here are cropped and the full-length blots/gels are presented in Additional file 2: Fig. S12. Supplementary Data 5. Quantification of immunostaining. We quantified the positive area of SIRT3 (A) and membranous GLUT1 (C) from indicated xenogr [file 12885_2020_6822_MOESM1_ESM.docx]

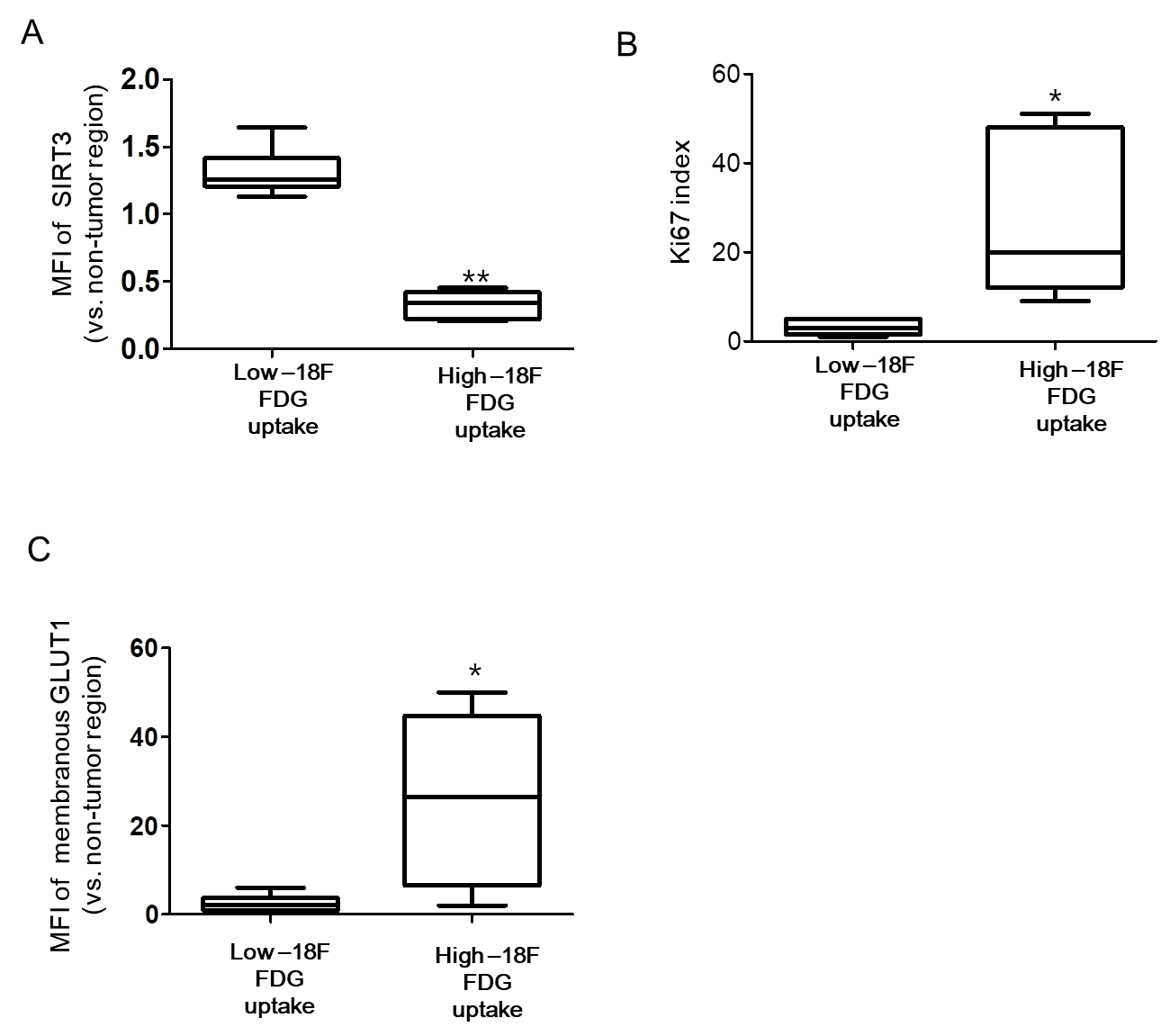


**Supplementary Data 1. Quantification of immunostaining.** (A, C) Mean fluorescent intensity (MFI) indicating the expression of (A) SIRT3 and membranous (C) GLUT1 from 12 patients with high FDG uptake (n=6) and low FDG uptake (n=6). Quantification of fluorescence in microscopic images stained with GFP (Green) and DsRed (Red) was carried out using IMT i-Solution software (Martin Microscope Company, Easley, USA). (B) Ki67 positive cells in positive and negative tumor regions of indicated proteins in HCC with high FDG uptake. Statistical analyses were performed using GraphPad Prism. Results are expressed as mean ± SD. Comparisons between groups were made using the Mann-Whitney test. **P* < 0.05; ***P* < 0.01.


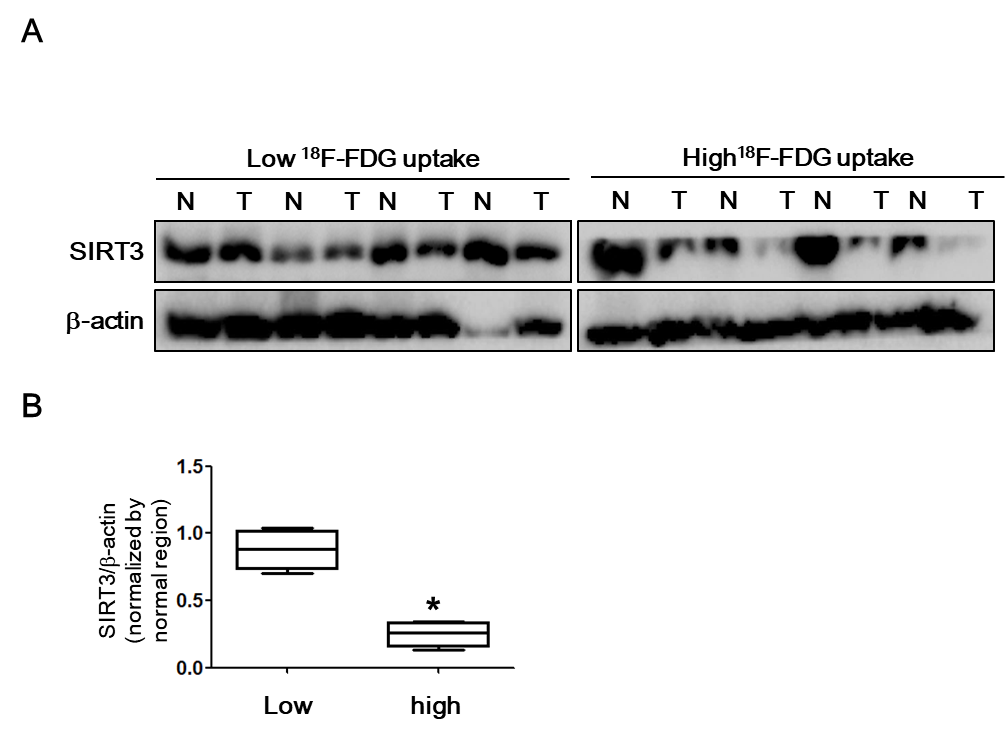


**Supplementary Data 2.**  SIRT3 expression in patients with hepatocellular carcinoma (HCC) and with different 18F-FDG uptake. (A) Protein was extracted from frozen HCC samples obtained after transsphenoidal surgery. Western blotting was performed using antibodies against SIRT3 and actin. The images shown here are cropped and the full-length blots/gels are presented in Additional file 2: Fig S11. (B) Band quantification was carried out using ImageJ. Statistical analyses were performed using GraphPad Prism. Results are expressed as mean ± SE. Comparisons between groups were made using the Mann-Whitney test. **P* < 0.05

**
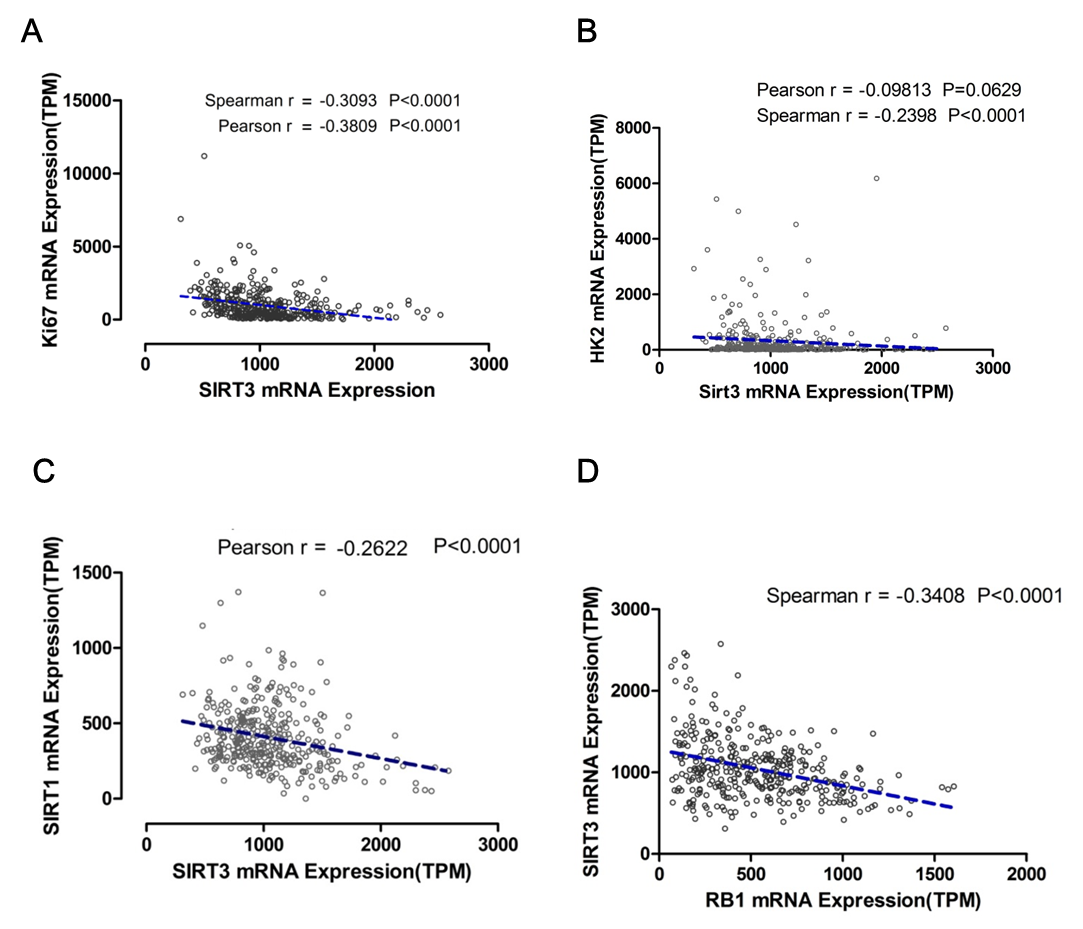
**

**Supplementary Data 3. TCGA data analysis.** The indicated mRNA level of the Cancer Genome Atlas (TCGA) Liver Hepatocellular Carcinoma data was obtained from OncoLnc (www.oncolnc.org) TCGA data portal.


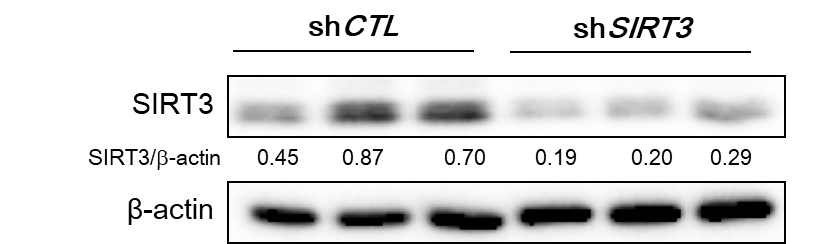


**Supplementary Data 4.**  SIRT3 expression in SIRT3 knockdown and control stable clones. The ratios of the band intensities were normalized by actin and are reported below the respective panels. The images shown here are cropped and the full-length blots/gels are presented in Additional file 2: Fig S12.

**
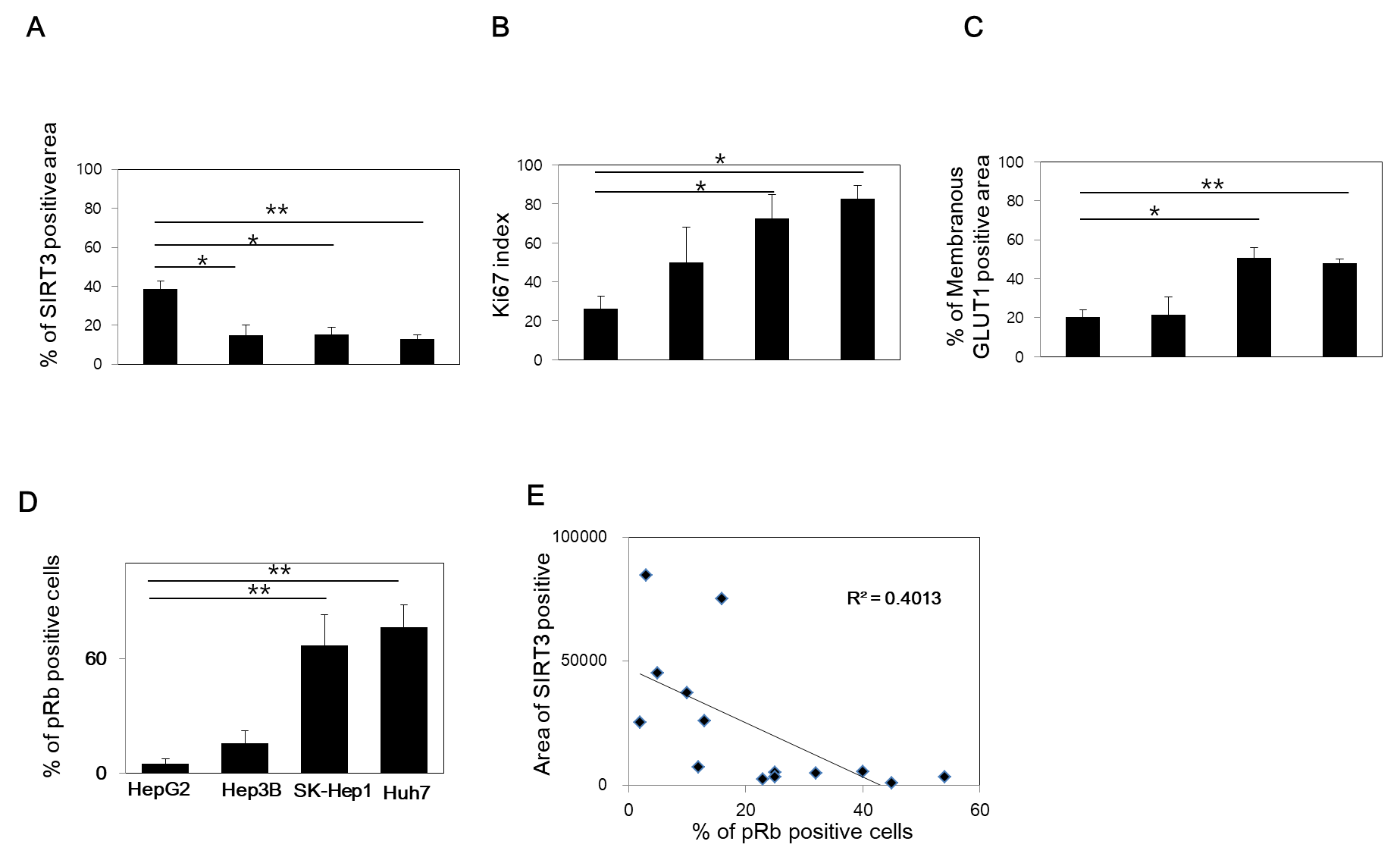
**

**Supplementary Data 5.**  Quantification of immunostaining. We quantified the positive area of SIRT3 (A) and membranous GLUT1 (C) from indicated xenograft model in Fig 2C. (B) We counted the Ki67 positive cells in tumor region of Fig 2C. (C) We quantified the positive cells of pRb from indicated xenograft model in Fig 4A. (D) Correlation of SIRT3 and pRb was performed in patients with HCC. Statistical analyses were performed using GraphPad Prism. Results are expressed as mean ± SE (range). Comparisons between groups were made using the Mann-Whitney test. *, P < 0.05;**, P < 0.01.

**
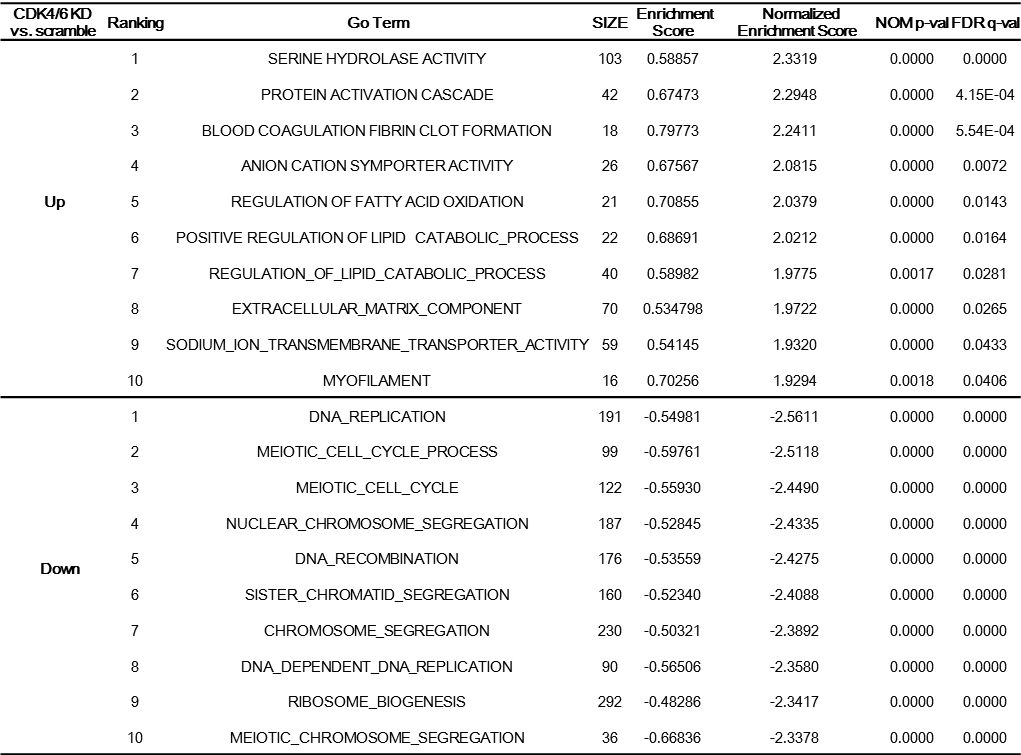
**

**Supplementary Data 6. Gene set enrichment analysis (GSEA)**

**
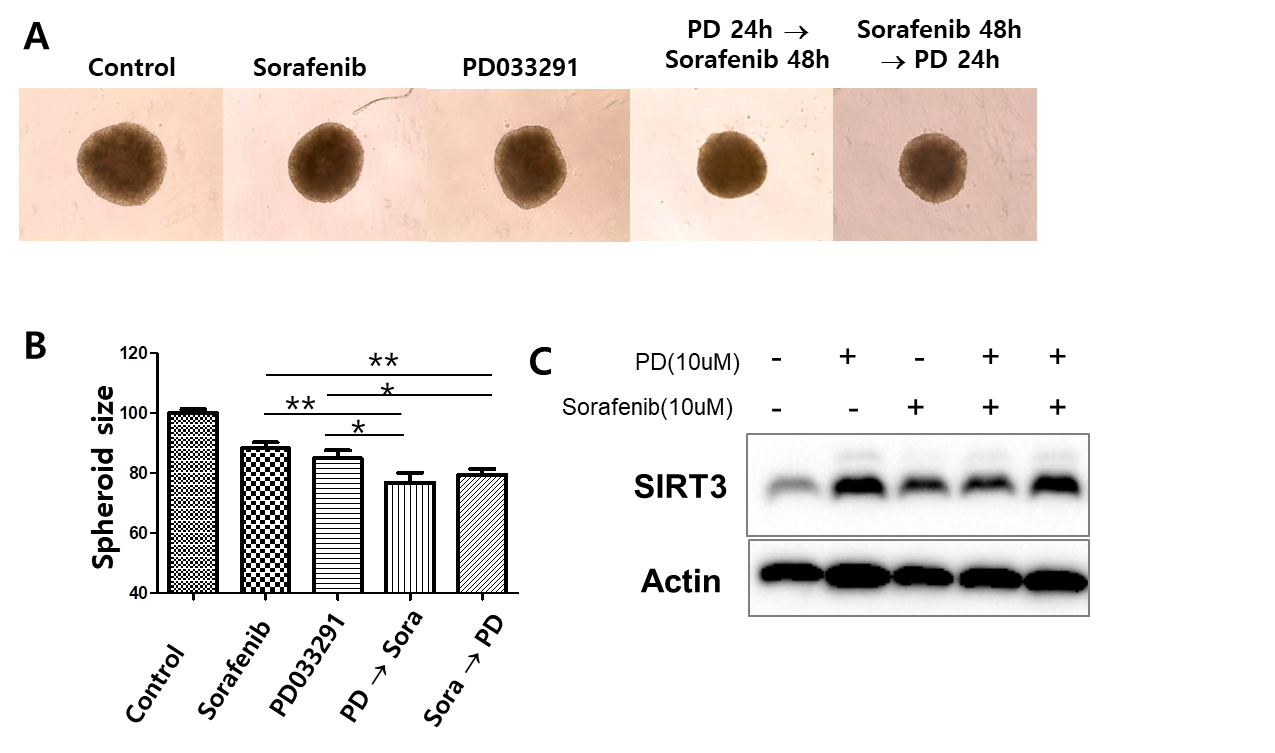
**

**Supplementary Data 7. The effect of combined treatment of sorafenib and PD033291 on spheroids from HepG2 cells.** (A) HepG2 cells were plated in low-affinity 96-well plates. After the formation of spheroids, HepG2 cells were treated with vehicle, 10 µM sorafenib, or PD033291 for the indicated incubation time. (B) Spheroid size was measured using Image J software after microscopy-based imaging based on six individual spheroids in each condition. Data are shown as the mean of three independent experiments ± SD. Data were analyzed using an unpaired t-test. *P < 0.05, **P < 0.01. (C) In parallel, western blotting was performed to detect SIRT3 and actin. The images shown here are cropped and the full-length blots/gels are presented in Additional file 2: Fig S13.

**
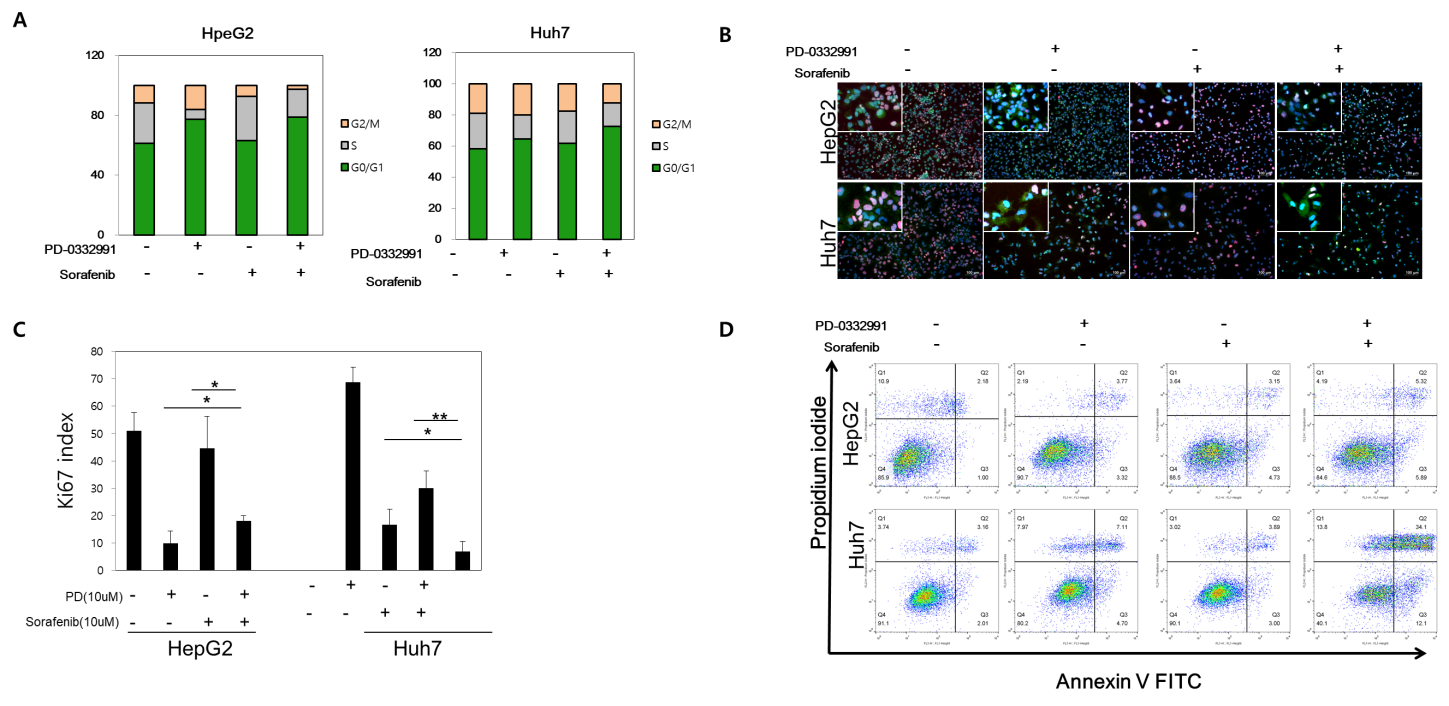
**

**Supplementary Data 8. Synergistic antitumoral effects of sorafenib and PD0332991**  (A) The cell cycle analysis after treatment with the combination of sorafenib and PD0332991 in HepG2 and Huh7 cells. (B) HepG2 and Huh7 were plated on coverslips in 24-well plates. The next day, the cells were incubated with indicated compound. After 24h, cells were fixed and processed for immunofluorescent staining with Ki67 protein and counterstained with DAPI. (C) In parallel, we counted Ki67 positive cells. (D) Analysis of apoptosis by Annexin V-APC/propidium iodide (PI) double staining of HepG2 and Huh7 cells. Two-color flow cytometry dot plots show the percentages of living cells as negative for both annexin V and PI; early-stage apoptotic cells as the populations testing Annexin V positive and PI negative, and late-stage apoptotic/necrotic cells as double-positive cells.

**
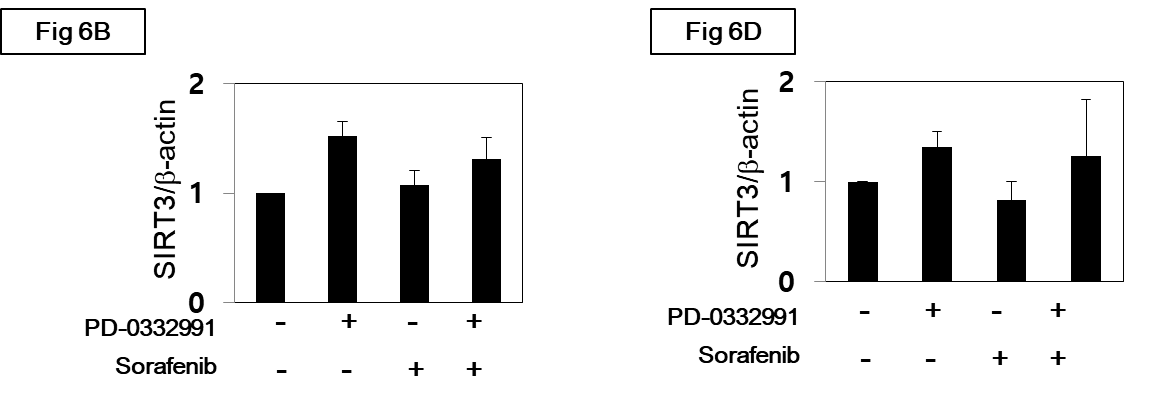

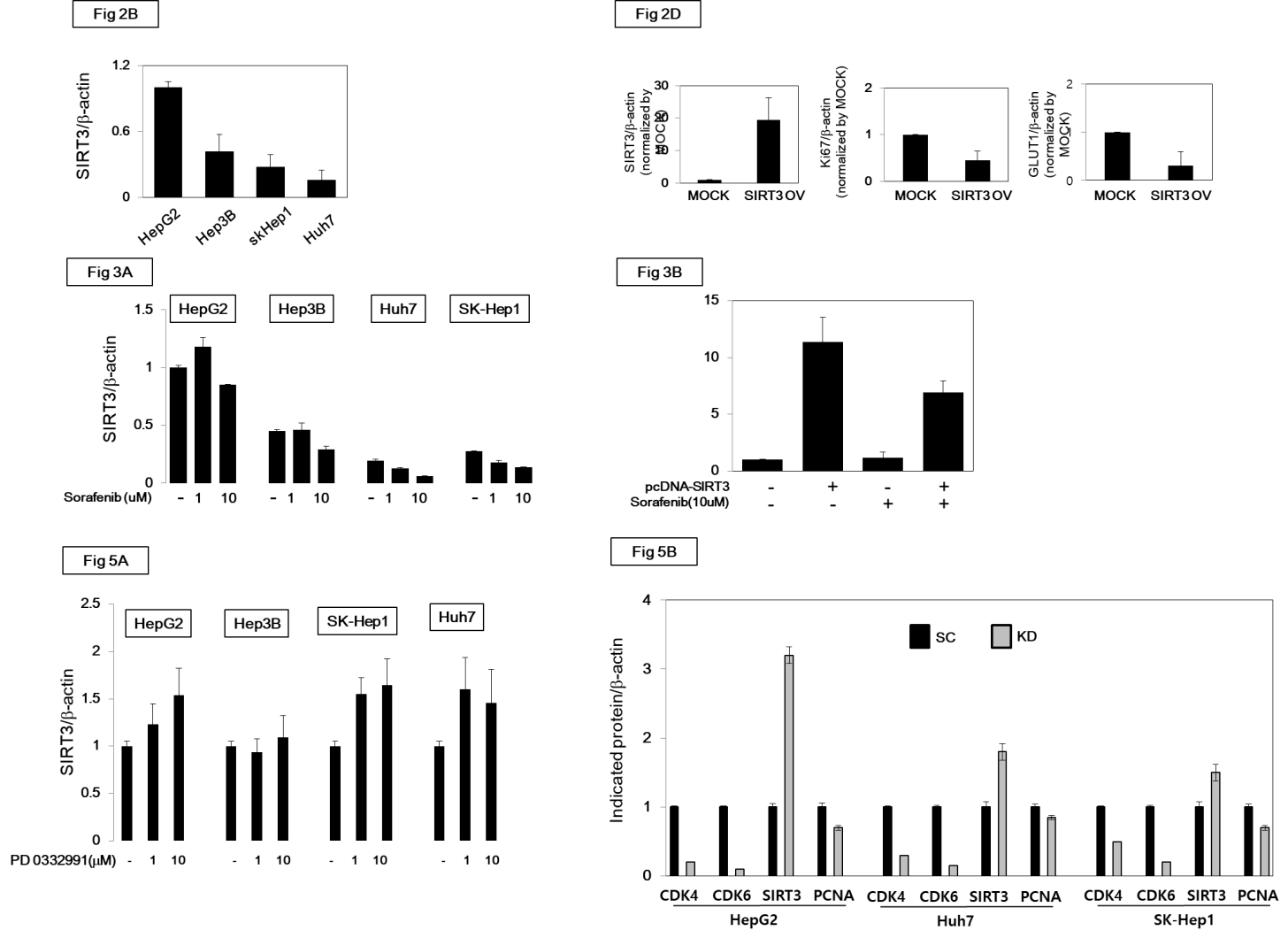
**

**Supplementary Data 9. Quantification of western blotting of Figures.**
