# Supplementary material for: Modulation of SIRT3 expression through CDK4/6 enhances the anti-cancer effect of sorafenib in hepatocellular carcinoma cells
Source: BMC Cancer. 2020 Apr 19;20:332. doi: 10.1186/s12885-020-06822-4 (PMC7168998; doi:10.1186/s12885-020-06822-4)
Supplement: Supplementary file 2 — Additional file 2 Figure S1. Full-length original blots of Fig. 2b. Figure S2. Full-length original blots of Fig. 2d. Figure S3. Full-length original blots of Fig. 3a. Figure S4. Full-length original blots of Fig. 3b. Figure S5. Full-length original blots of Fig. 5a. Figure S6. Full-length original blots of Fig. 5b (HepG2). Figure S7. Full-length original blots of Fig. 5c(Huh7). Figure S8. Full-length original blots of Fig. 5d (SK-Hep1). Figure S9. Full-length original blots of Fig. 6b. Figure S10. Full-length original blots of Fig. 6d. Figure S11. Full-length original blots of Supporting data 2. Figure S12. Full-length original blots of Supporting data 4. Figure S13. Full-length original blots of Supporting data 7. [file 12885_2020_6822_MOESM2_ESM.pdf]

- We used ChemiDoc XRS (Biorad), which enables direct digital visualization of chemiluminescent western blots for the image of signals accumulated in the chemiluminescence reaction.

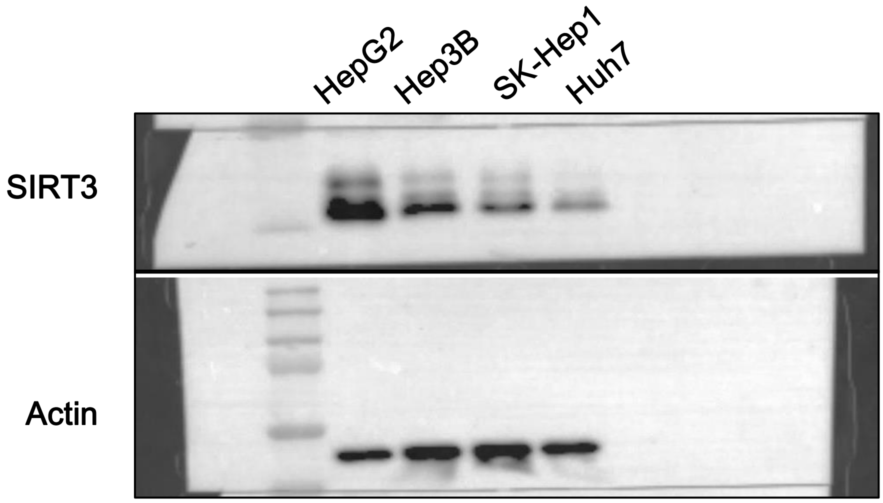

Fig S1. Full-length original blots of Fig2B.

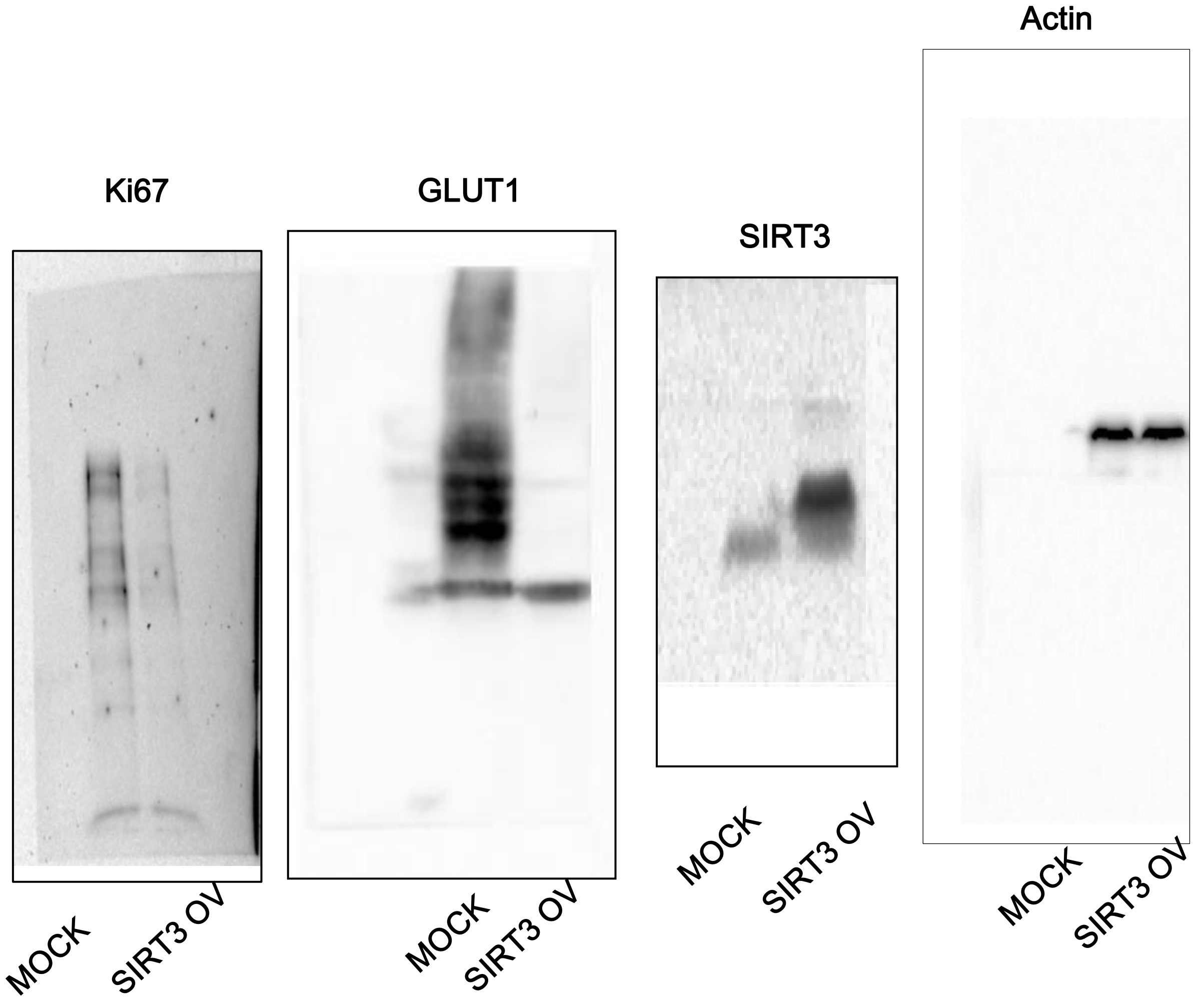

Fig S2. Full-length original blots of Fig2D.

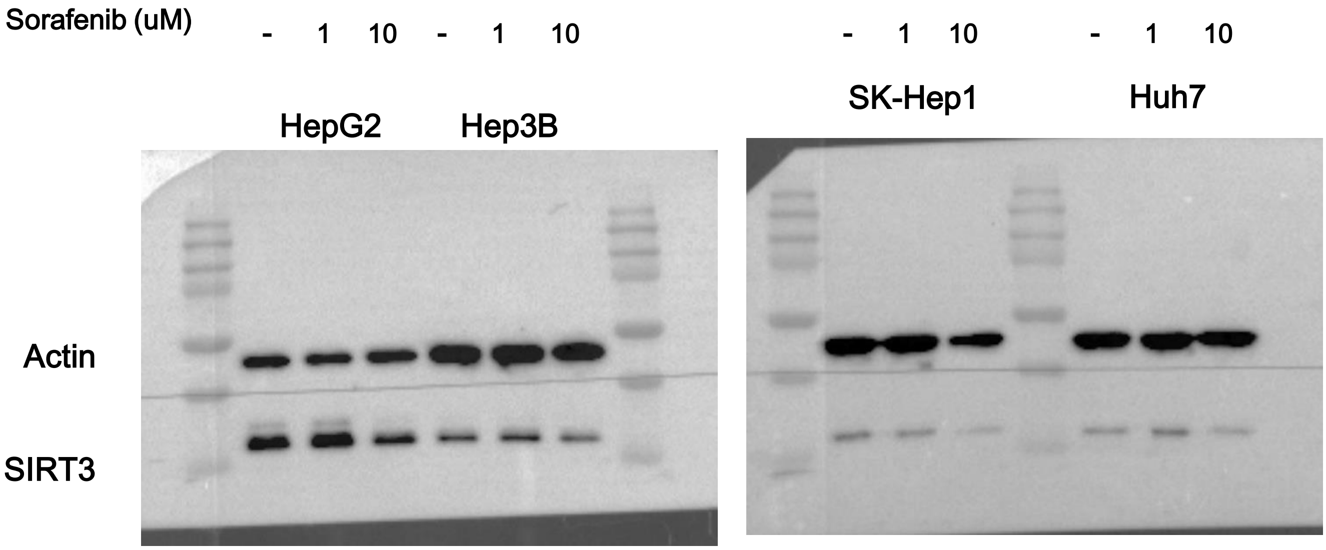

Fig S3. Full-length original blots of Fig 3A.

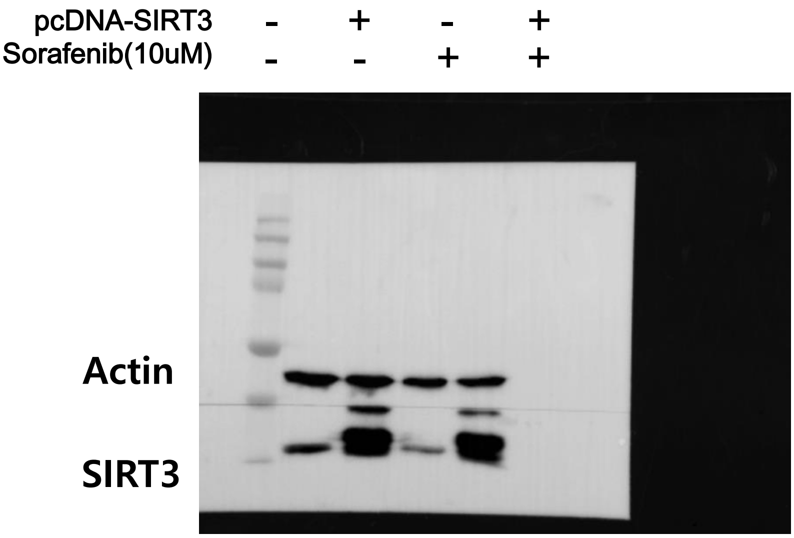

Fig S4. Full-length original blots of Fig3B

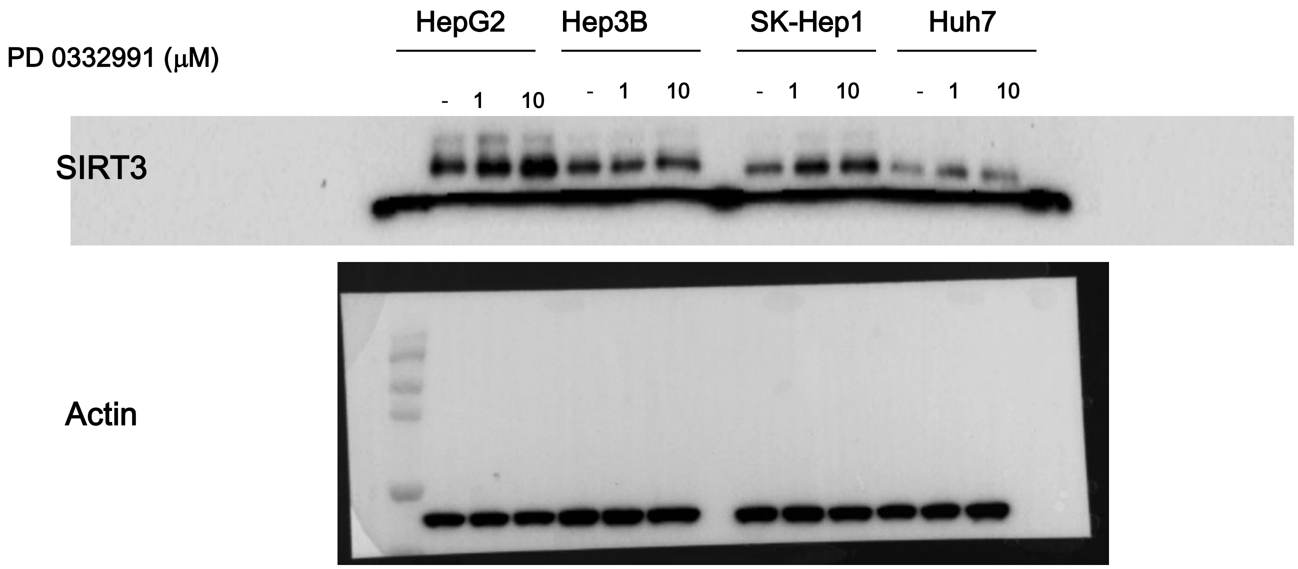

Fig S5. Full-length original blots of Fig 5A

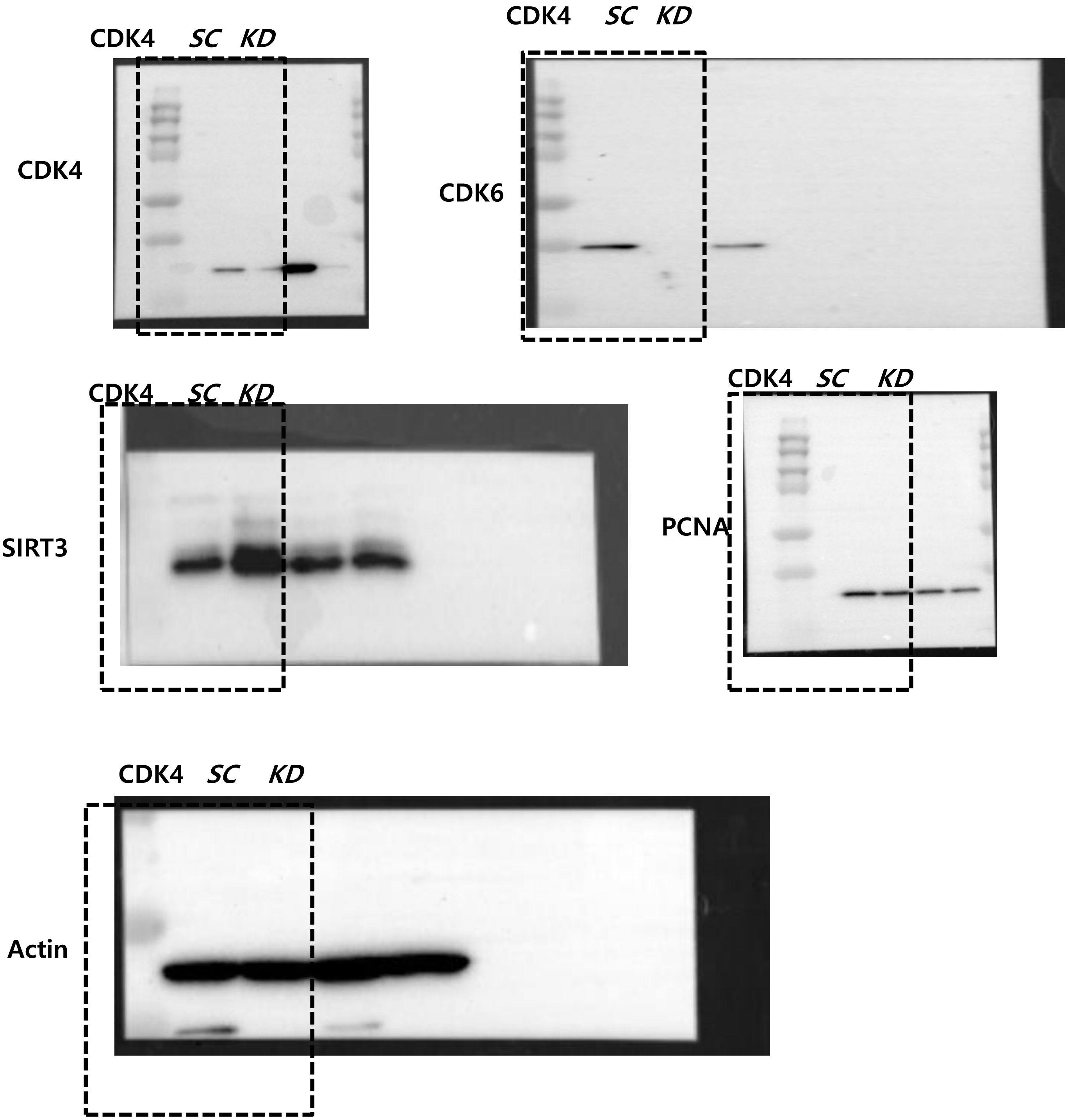

Fig.S6 Full-length original blots of Fig 5B (HepG2).

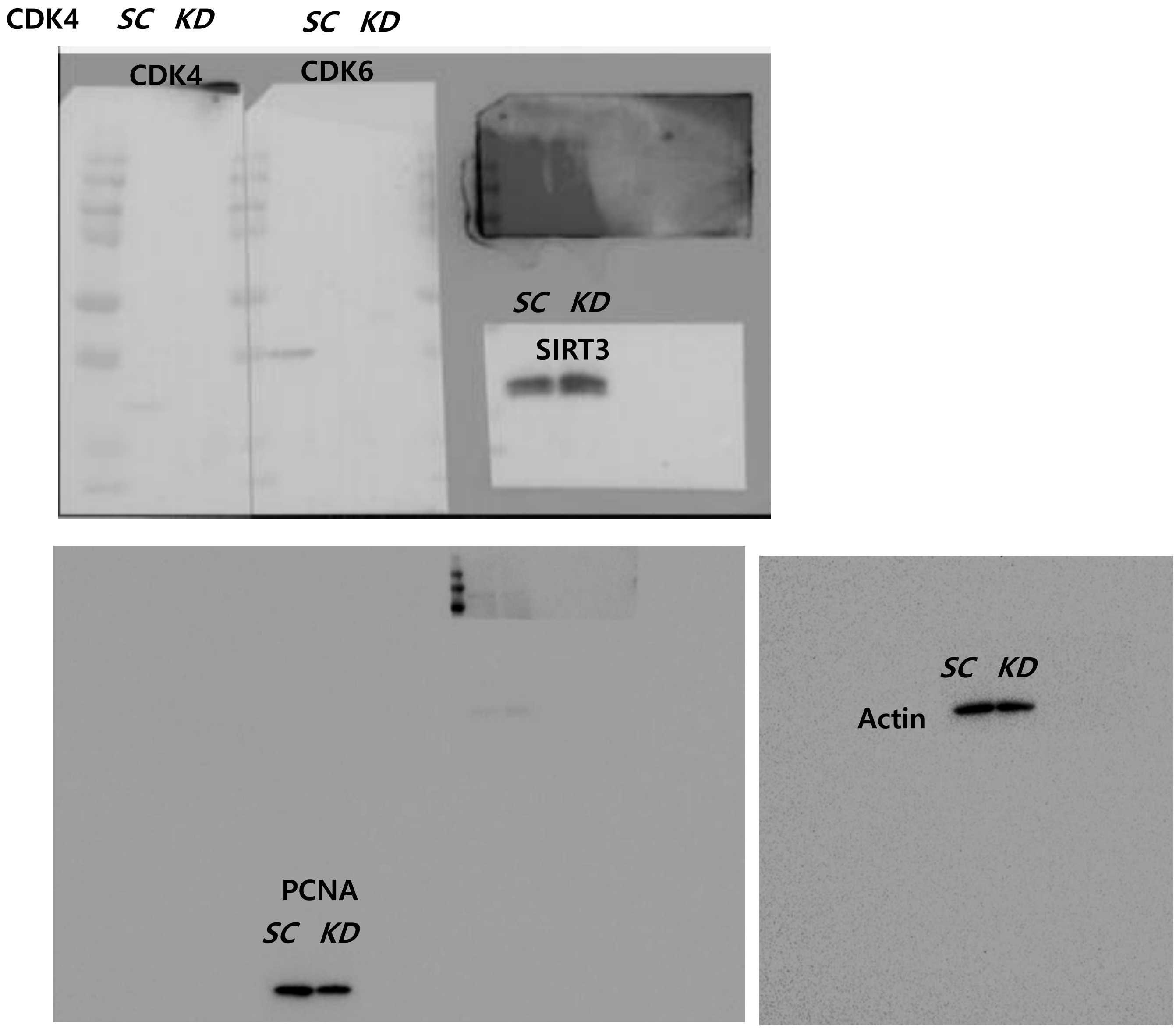

Fig S7. Full-length original blots of Fig 5C(Huh7).

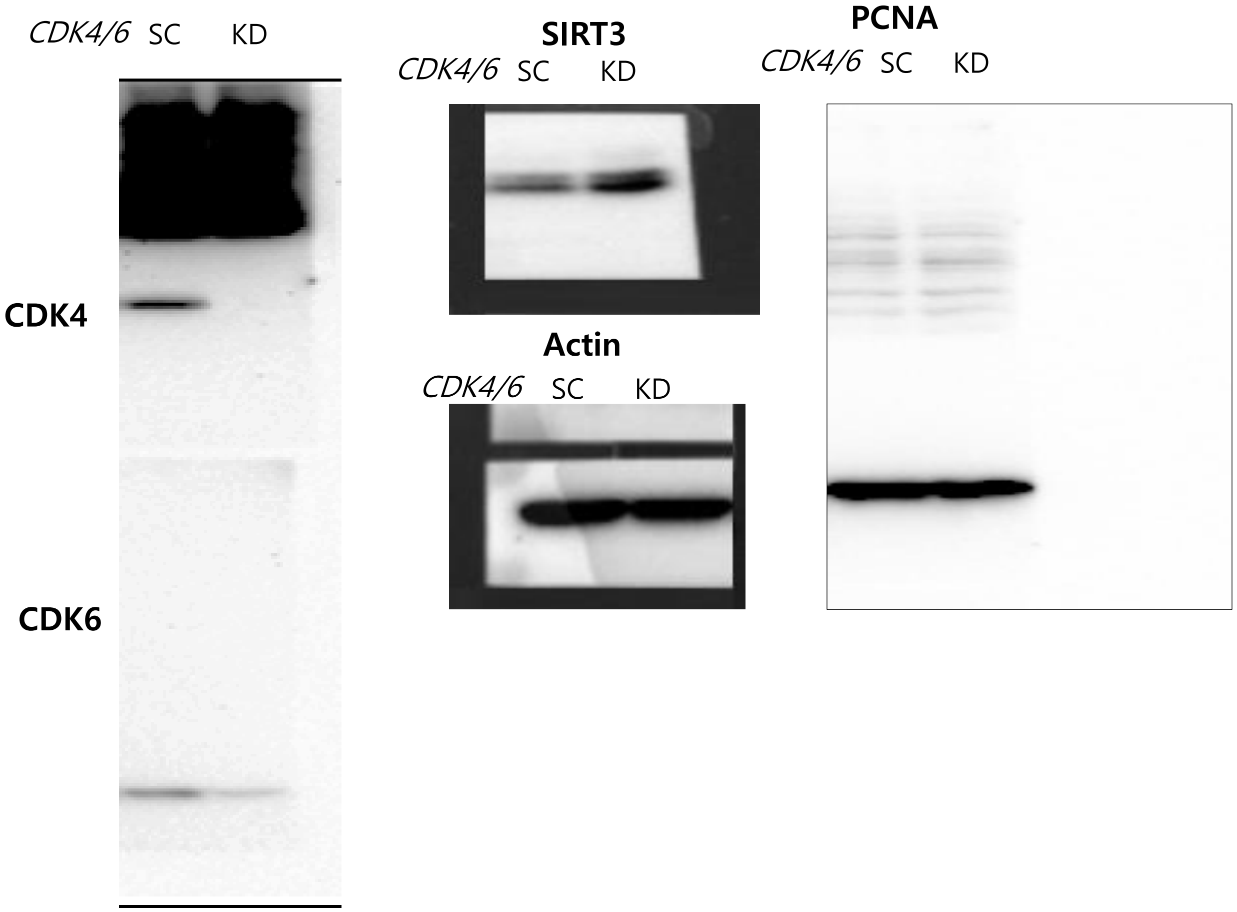

Fig S8. Full-length original blots of Fig 5D (SK-Hep1).

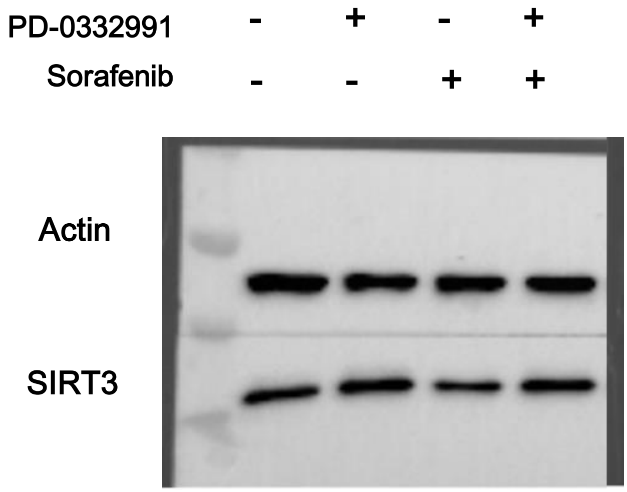

Fig.S9 Full-length original blots of Fig 6B

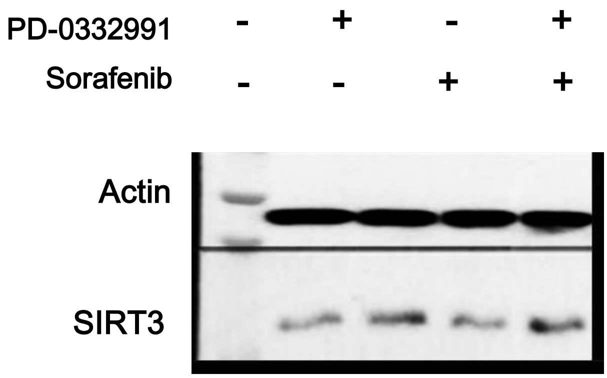

Fig.S10 Full-length original blots of Fig 6D

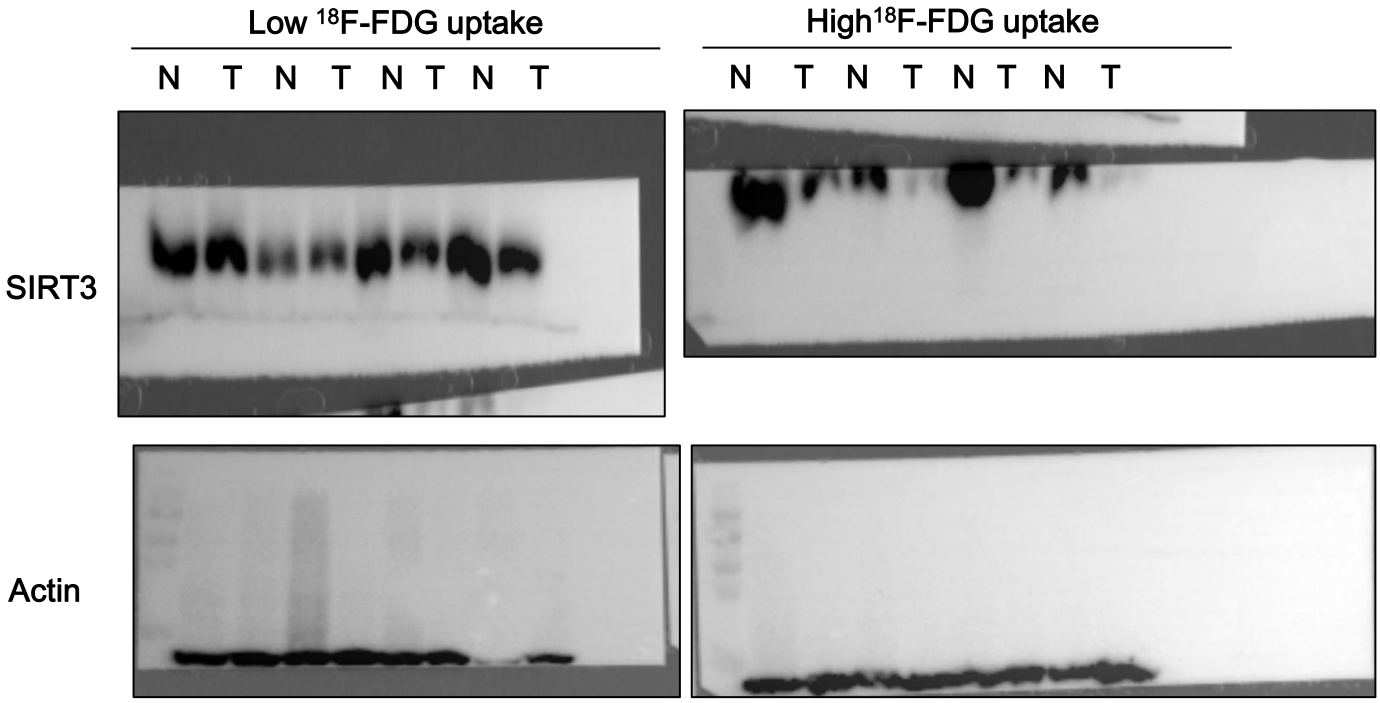

Fig.S11 Full-length original blots of Supporting data 2.

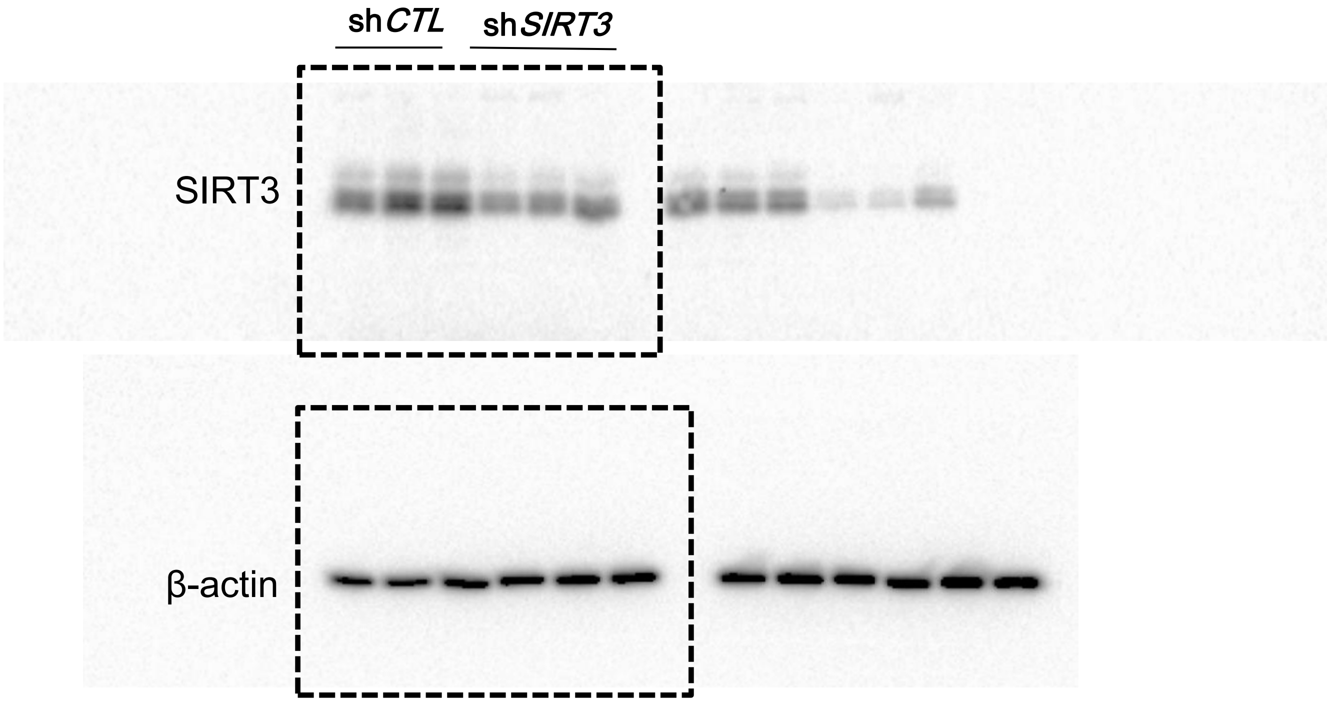

Fig. S12. Full-length original blots of Supporting data 4.

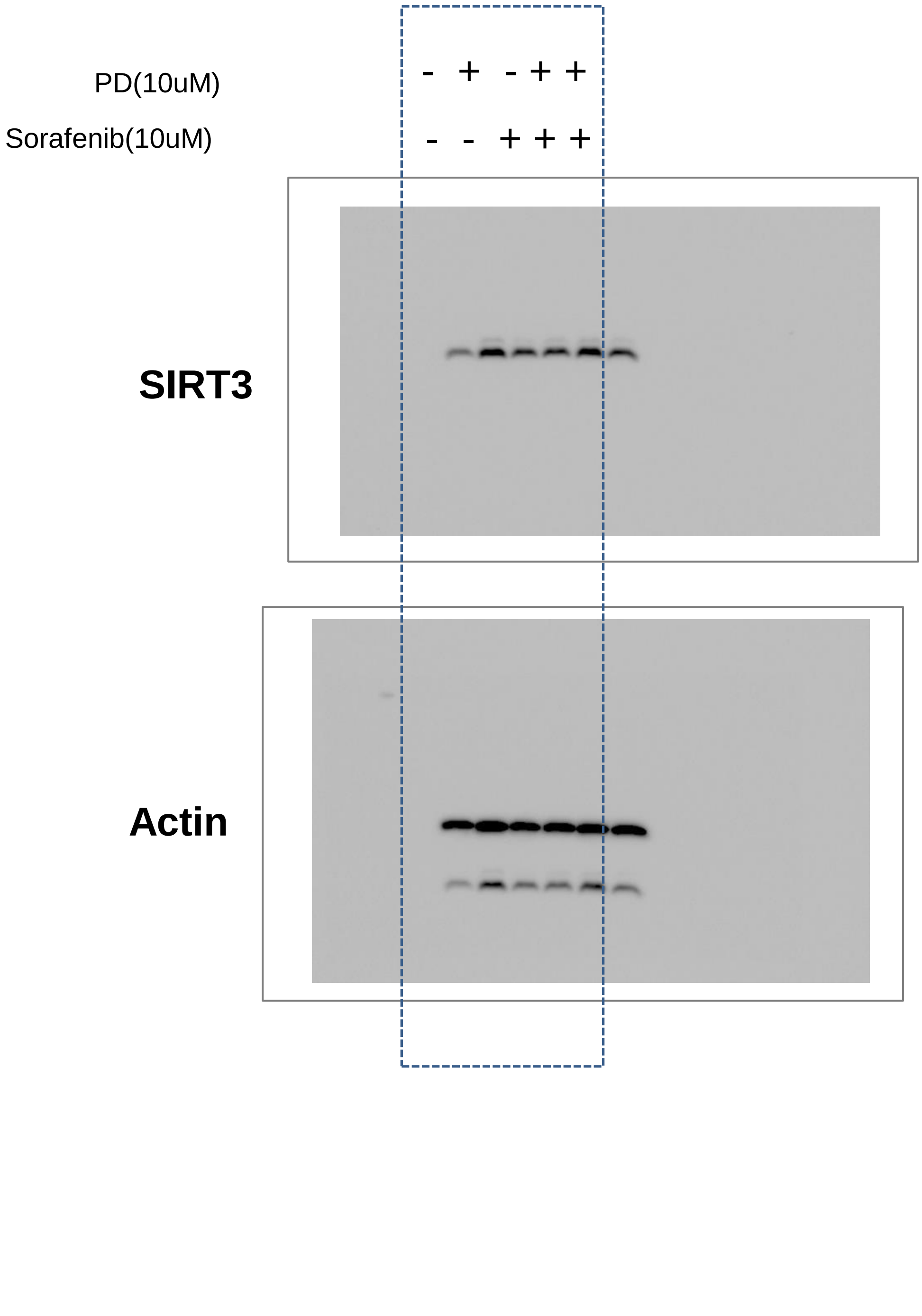

Fig. S13. Full-length original blots of Supporting data 7.
